# The Accuracy of Diagnostic Tests for Lyme Disease in Humans, A Systematic Review and Meta-Analysis of North American Research

**DOI:** 10.1371/journal.pone.0168613

**Published:** 2016-12-21

**Authors:** Lisa A. Waddell, Judy Greig, Mariola Mascarenhas, Shannon Harding, Robbin Lindsay, Nicholas Ogden

**Affiliations:** 1 National Microbiology Laboratory, Public Health Agency of Canada, Guelph, Ontario, Canada; 2 National Microbiology Laboratory, Public Health Agency of Canada, Winnipeg, Manitoba, Canada; 3 National Microbiology Laboratory, Public Health Agency of Canada, St. Hyacinthe, Quebec, Canada; University of Kentucky College of Medicine, UNITED STATES

## Abstract

There has been an increasing incidence of Lyme disease (LD) in Canada and the United States corresponding to the expanding range of the Ixodes tick vector and Lyme disease agent (*Borrelia burgdorferi sensu stricto)*. There are many diagnostic tests for LD available in North America, all of which have some performance issues, and physicians are concerned about the appropriate use and interpretation of these tests. The objective of this systematic review is to summarize the North American evidence on the accuracy of diagnostic tests and test regimes at various stages of LD. Included in the review are 48 studies on diagnostic tests used in North America published since 1995. Thirteen studies examined a two-tier serological test protocol vs. clinical diagnosis, 24 studies examined single assays vs. clinical diagnosis, 9 studies examined single immunoblot vs. clinical diagnosis, 7 studies compared culture or PCR direct detection methods vs. clinical diagnosis, 22 studies compared two or more tests with each other and 8 studies compared a two-tiered serological test protocol to another test. Recent studies examining the sensitivity and specificity of various test protocols noted that the Immunetics® C6 *B*. *burgdorferi* ELISA™ and the two tier approach have superior specificity compared to proposed replacements, and the CDC recommended western blot algorithm has equivalent or superior specificity over other proposed test algorithms. There is a dramatic increase in test sensitivity with progression of *B*. *burgdorferi* infection from early to late LD. Direct detection methods, culture and PCR of tissue or blood samples were not as sensitive or timely compared to serological testing. It was also noted that there are a large number of both commercial (n = 42) and in-house developed tests used by private laboratories which have not been evaluated in the primary literature.

## Introduction

Lyme disease (LD) is the most common tick-borne infection in North America [1,2]. It was first publically recognized in the United States in 1975 in the towns of Lyme and Old Lyme Connecticut as a result of an investigation into 51 cases (39 children) with a similar form of arthritis, although the first case was describe five years earlier by a dermatologist in Wisconsin [3,4]. In North America early signs of infection may include erythema migrans (EM, a characteristic skin rash that often has a bulls eye appearance) and fever and non-specific symptoms like headache and lethargy [5,6]. If untreated, the disease can progress to disseminated LD with neurological, cardiac and arthritic manifestations [7]. Lyme disease in North America is caused by *Borrelia burgdorferi sensu stricto* (hereafter called *B*. *burgdorferi*) and recently *Borrelia mayonii* was identified and may be responsible for a proportion of cases, however the performance of LD diagnostic tests to identify *B*. *mayonii* infection is not available [8]. In Europe *B*. *afzelii*, *B*. *garinii*, *B*. *burgdorferi*, *B*. *spielmanii*, *B*. *bissettii* and *B*. *bavariensis* cause disease with a wider variety of symptoms than reported in North America; a number of genospecies including *B garinii* occur in Asia.

Ticks of the genus *Ixodes* transmit the spirochete when they feed. *Ixodes scapularis*, the blacklegged tick, is the main vector in northeastern and upper midwestern United States and Canada while *I*. *pacificus* is the major vector in western United States and western Canada [9,10]. The primary vectors of LD in Europe and Asia are *I*. *ricinus* and *I*. *persulcatus* respectively [6,11]. The principal natural hosts of immature stages of the ticks and *B*. *burgdorferi* include rodents, other small and medium sized mammals, reptiles and birds, while adult female ticks feed mainly on deer [12].

Lyme disease incidence has increased since 1975 as the tick vectors have expanded their geographic range across the north eastern and upper mid-western states in the US and more recently into Canada [2,13]. Range and spread of ticks and *B*. *burgdorferi* is facilitated by migratory birds and terrestrial hosts [14]. There is increasing evidence that climate change will result in further northward expansion of the tick vector’s range in Canada, resulting in increased future risk of LD among Canadians [15,16].

The diagnostic tests available for confirmation of human LD have variable sensitivity and specificity depending on the stage of infection, thus it is important to monitor the literature on available tests for LD to promote those tests that perform the most effectively and address concerns about the performance of non-validated tests and test protocols using evidence-informed strategies for decision making [17,18]. Currently in Canada and the United States, a two-tiered serology protocol is the only validated diagnostic approach for LD diagnosis recommended by United States CDC and the Public Health Agency of Canada [17,18]. This two-tiered test is typically an enzyme immunoassay (EIA) to detect IgM or IgG antibodies to *B*. *burgdoferi* in serum and if the sample is positive or equivocal on the screening assay, then a western blot is used to detect serum IgM or IgG antibodies to *B*. *burgdorferi*. Use of IgM testing is recommended during the first 30 days of infection, after which only IgG tests should be used. Currently, only serology tests have been licensed for use by the FDA and the Health Canada Medical Devices Branch (HC) for LD testing [19,20]. Other direct detection tests such as PCR may be commercially available, but they have not been licensed for use by a governing body. There are a number of commercial EIA kits that are licensed by the FDA and/or HC and use either whole cell preparation of *B*. *burgdorferi* and/or purified recombinant or chimeric antigens (see S2 Text). Other EIAs reported in the literature have been developed within the reporting laboratory and have not been commercialized or under-gone licensing and will be referred to as in-house developed tests [21,22]. The EIA’s have good sensitivity after 30 days of infection, but typically suffer from lower specificity [22]. In 1995, the Centers for Disease Control and Prevention (CDC) adopted criteria for interpreting the results of the western blot for LD and most commercialized tests follow these guidelines [23].

The objective of this systematic review is to summarize the North American evidence on the accuracy of diagnostic tests and test regimes used to diagnose LD in patients presenting with clinical symptoms in North America at various stages of disease and to address the question of whether there is evidence of superior, equivalent or poor performance by the commercial (approved by the FDA and/or HC) and in house laboratory tests captured in this review. To the best of our knowledge this systematic review is a significant update to Dumler (2001) [24] and is complementary to a recent systematic review on European Lyme disease diagnostic tests [25].

## Methods

### Scoping review

This systematic review was preceded by a scoping review conducted by Greig et al (2016) to identify, classify and characterise what is the current state of scientific knowledge on surveillance methods, prevention and control strategies, diagnostic tests, risk factors, and societal attitudes and perceptions towards LD in humans and *B*. *burgdorferi* in tick vectors and vertebrate reservoirs [26]. Briefly, the scoping review methodology was designed to characterise the primary literature on LD in humans or *B*. *burgdorferi* tick vectors or reservoirs, thus studies not on LD or *B*. *burgdorferi* were excluded from the scoping review. Additionally, the primary research had to address one of the following topics: surveillance/monitoring, prevalence, incidence, societal attitudes and perceptions in North America and global prevention and control strategies, diagnosis and risk factors. Research on clinical LD and treatment were considered outside the scope of this review. Each relevant paper was classified by purpose, study design, location of the study, *B*. *burgdorferi*, host species investigated, vector species investigated, sampling dates, diagnostic tests used, and whether the paper contained extractable data.

The scoping review search strategy was developed and pretested by three individuals with extensive experience in knowledge synthesis, zoonotic diseases and library science. The following search algorithm was implemented in eight bibliographic databases: BIOSIS (via web of knowledge), CAB abstracts, Scopus, PubMed, PsycINFO, APA PsycNet, Sociological Abstracts, and EconLit with no limitation on the search, this was followed by a comprehensive search for grey literature [26]: (lyme OR borrelia) AND ("host" OR sentinel OR landscaping OR "vector" OR "vectors" OR "monitor" OR "monitoring" OR surveillance OR reservoir OR reservoirs OR prevalence OR educate OR education OR barrier OR barriers OR intervene OR intervention OR incidence OR rate OR prevent OR prevention OR control OR risk OR risks OR attitude OR attitudes OR perception OR perceptions OR diagnostic). The search was conducted September 13^th^-14^th^, 2013 and no update of the search has been performed as analysis indicated the findings would not change with the addition of new papers, thus the resources required to conduct the update were not prioritized. The protocol for the scoping review is available upon request.

### Systematic review methods

Studies identified in the scoping review that evaluated diagnostic tests for humans were fully evaluated in this systematic review. The systematic review tools include a confirmation of relevance, location of study, availability of extractable data and a quality assessment form based on the Quality Assessment of Diagnostic Accuracy Studies (QUADAS-2) tool [27–29]. This tool assesses the risk of bias and other methodological quality domains to evaluate the extent to which the results of each study or group of studies could be biased. The QUADAS-2 tool assessed the four quality domains (Table 1) with respect to patient selection, the diagnostic tests used, the reference standard and flow and timing of the study [28]. An additional section was added to evaluate comparison tests and capture the presence of funding bias [30,31].

**Table 1:** Number of studies meeting each quality criteria in QUADAS-2 based on 48 articles from the United States examining the accuracy of diagnostic tests for Lyme disease included in this systematic review.

**Assessment Question**	**Yes/Low**	**Unclear**	**No/ High**	**NA/NR**
**Domain 1: Patient Selection**				
**Was a consecutive or random sample of patients enrolled?**	12	34	2	
**Was a case-control design avoided?**	43	4	1	
**Did the study avoid inappropriate exclusions?**	41	6	1	
**Was there a Risk of Bias (RoB) due to patient selection?**	18	31	0	
**Is there concern that the included patients do not match the review question? (applicability)**	39	8	1	
**Domain 2: Index Test = Two-tier method**				
**Were the index test results interpreted without knowledge of the results of the clinical reference standard?**	8	12	0	
**If a threshold was used, was it pre-specified?**	19	1	0	
**Could the conduct or interpretation of the index test have introduced bias? (RoB)**	10	10	0	
**Is there concern that the index test, its conduct or interpretation differs from the review question? (applicability)**	19	1	0	
**Domain 3: Clinical Reference Standard (Clinical diagnosis)**				
**Is the clinical reference standard likely to classify the target condition correctly?**	35	13	0	
**Is there undue increased RoB on the described physician evaluation of the patients included in this study?**	36	12	0	
**Domain 4: Flow and Timing**				
**Is the time period between the clinical reference standard and the index test/other tests appropriate to be reasonably sure the target condition did not change between the two tests?**	47	1	0	
**Did all patients receive the same clinical reference standard?**	41	7	0	
**For studies with multiple comparator tests, was the whole sample or a random selection of samples used to define which patients were tested with a particular test?**	37	1	1	9
**Were all participants included in the analysis?**	42	5	1	
**Could the flow or timing of the study execution have introduced bias? (RoB)**	48	0	0	
**Domain 5: Comparison Tests**				
**Were the comparison tests interpreted without knowledge of the results of the index test?**	17	27	2	
**If a threshold was used, was it pre-specified?**	43	1	2	
**Could the conduct or interpretations of the comparison test(s) have introduced bias? (RoB)**	27	19	0	
**Is there concern that the comparison test(s), tis conduct or interpretation differs from the review question? (applicability)**	42	4	0	
**Additional Bias Questions**				
**Was inappropriate variation in the results by technician, laboratory or instruments reported?**	15	2	1	30
**Was the study free of commercial funding or are we confident the results were not influenced by a commercial enterprise?**	35	9	4	
**Overall Risk of Bias (RoB)**	8	40	0	

The data extraction form captured all pertinent study details and results. The systematic review was managed in DistillerSR (Evidence Partners, Ottawa, ON, Canada) a web-based systematic review management software. Each form was completed by two reviewers working independently and conflicts were resolved by consensus. Data were exported to Microsoft Excel 2010 (Microsoft Corp., USA), prepared for summarization and analysed in STATA v. 13 (StataCorp., USA). The study protocol and PRISMA evaluation can be found in the supplementary material (S1 Text, S3 Text).

Included papers examined the accuracy of diagnostic tests for LD in North America after 1995, and included studies that compared results of one test using a validated test panel, results of clinical diagnosis, or a gold standard test result or investigated inter-test agreement. The recommendations for two-tier testing occurred in 1995, so we limited the review to studies conducted after 1994. Studies that screened an asymptomatic population for LD were excluded from this study. No inclusion or exclusion criteria were implemented on the type of control group; instead it was evaluated as a source of variation between study results (heterogeneity). The control group was usually a mix of one or more categories of healthy volunteers from non-LD endemic or LD endemic regions, or asymptomatic blood donors. In some studies, patients with diseases that have similar signs and symptoms to LD or have humoral responses that overlap with LD and are known to cross-react (e.g. rheumatoid arthritis, systemic lupus erythematosus, syphilis, autoimmune disorders, leptospirosis, periodontitis, relapsing fever, tularemia, Southern Tick-associated Rash Illness (STARI), multiple sclerosis, and Epstein-Barr virus infection) were included as controls to more precisely define test specificity. Studies often used well-defined samples from serum repositories or panels, like those developed by CDC [32], a research institute [33,34] or a commercial company [35]. These results were included in this systematic review and the impact of patient-based or panel samples on the outcome was investigated.

For this review the stages of LD are as follows: Early / acute LD (stage 1) is defined as those patients presenting with EM and/or associated manifestations that have experienced signs and symptoms of LD for less than 30 days [7]. Stage 2 illness is early disseminated LD, which includes manifestations of early neurological LD, cardiac LD and multiple EMs [36]. Stage 3 is late LD, typically with manifestations of Lyme arthritis and late neurological LD [36]. Those patients tested after antibiotic therapy are described as convalescent with the stage of LD assigned prior to treatment. Post treatment Lyme syndrome is defined as a condition where despite treatment the patient continues to experience illness [37]. “Chronic LD” is a condition that is not recognised as being caused by *B*. *burgdorferi* by most infectious disease experts, occurs in patients exhibiting non-specific illness who do not test positive on Food and Drug Administration (FDA) approved serological tests, so these have been excluded from this review [38].

### Meta-Analytic Methods

The dataset was managed in MS excel; each line of data represents a single test accuracy outcome and one study may have several comparisons, thus several lines of data. Each comparison was extracted, grouped and coded according to tests and type of outcome reported. When there were four or more lines of data for a category, meta-analysis was conducted using hierarchical logistic regression and bivariate models in Stata 13 using Metandi and Midas command packages. These models have been designed to account for the correlation between sensitivity and specificity [39] and they overcome the often violated assumptions of a linear regression model [40,41]. These hierarchical models use 2x2 cell counts to compute log transformations of proportions for the analysis [39]. Without covariates, the hierarchical summary receiver operating characteristic (HSROC) and bivariate models are equivalent although their assumptions are different: HSROC assumes there is an underlying Receiver-Operating Characteristic (ROC) for each study and the bivariate model directly models the log-odds transformed sensitivity and specificity assuming a bivariate normal distribution between studies [42].

Meta-analytic statistical summaries of sensitivity, specificity, likelihood ratios and diagnostic odds ratio have been summarized where possible in the tables. Model diagnostics including goodness of fit, normality, influential and outlying points, publication bias and heterogeneity were examined where possible. Publication bias was not evaluated when heterogeneity was >60% or there were less than 10 lines of data. Meta-regression using the bivariate model was used to examine whether predetermined covariates explain some of the between-study variation given there was sufficient data to fit the model (>10 data lines per covariate).

## Results

**Fig 1:**
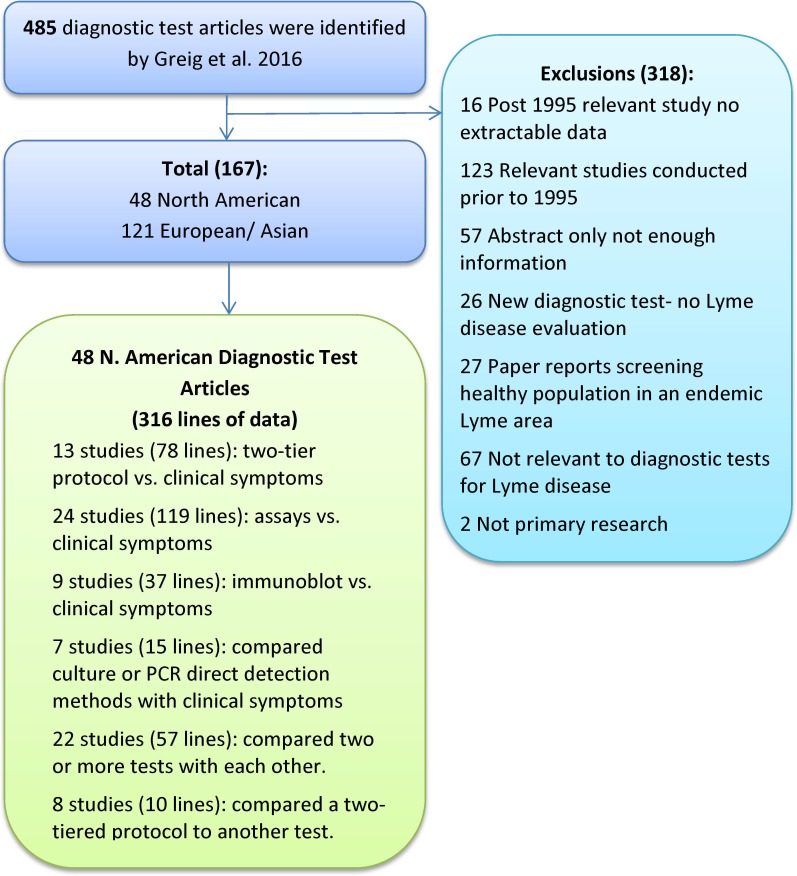
Flow diagram of diagnostic test papers through the systematic review from the scoping review.

In the scoping review, 485 articles focused on diagnosis of LD in humans globally and were further evaluated for inclusion in this systematic-review meta-analysis. The decision tree for selection of articles and reasons for exclusion of potentially relevant studies in this systematic review is shown in Fig 1. Forty-eight relevant diagnostic test evaluations conducted in North America between 1995 and 2013 were included in this systematic review (see S2 Text and S1 Dataset).

The QUADAS-2 tool results, Table 1, indicated that there was an unclear risk of bias in 84% of studies, meaning the study received an unclear or high risk of bias score on one or more domains (see S1 Dataset). No studies were excluded from the analysis based on their QUADAS assessment. In two studies it was apparent that the sample population was not appropriately enrolled in the study as the case population and control population were enrolled at different times and places, which could lead to biased (exaggerated) results for test accuracy [43,44]. Appropriate blinding was often not addressed in many papers and unexplained exclusion of observations from the analysis was another common reporting issue. Many of the studies (28.6%) had authors employed by or funded by commercial companies that supplied one or more of the tests evaluated. In four of these studies the risk of funding bias was identified to be very high [43,45–47].

### The diagnostic accuracy of tests compared to clinical diagnosis

#### Two-tier serological test vs. clinical diagnosis

Thirteen studies evaluated the two-tier serological test protocol for diagnosis of LD at different stages of disease and after antibiotic therapy. Table 2 provides the meta-analytic summaries demonstrating low sensitivity, 46.3% (95%CI 39.1–53.7), for early (stage 1) LD patients and increasing sensitivity with stage 2, 89.7% (78.3–95.4), and stage 3, 99.4%^H^ (95.7–99.9) LD. There was relatively high specificity (98.3%–99.9%) across control groups. Most false positives within the control groups were patients with diseases known to produce antibodies that cross-react in serological tests for *B*. *burgdorferi*. Nine studies (14 lines of data) presented results for two-tier serological testing where at least one of the tests was not FDA licensed (designed in house by the reporting laboratory), Table 2. Heterogeneity analysis of sensitivity and specificity on the impact of using non-commercial tests was not significant. At the early stage of LD the two-tier testing method was good for ruling in LD if the patient tested positive, but had very poor predictive value for ruling out LD, which is why it is recommended to retest after 30 days [21]. However, for convalescent patients treated at stage 1 LD sensitivity remained low even after 30 days.

**Table 2:** Thirteen studies (78 lines of data) evaluating a two-tier serological test protocol summarized by the stage of Lyme disease tested using a hierarchical logistic regression model in Stata 13 or the range of sensitivity and specificity data presented in the paper when less than four lines of data were available for meta-analysis.

Description- stage LD	Studies (lines)	Sn estimate (95% CI)	Sp estimate (95% CI)	LR+	LR-	DOR
All Stages of LD*	7 (11)	57.6% (49.4–65.4)	99.2% (98.3–99.6)	69.5	0.43	162.6 (80.8, 327.3)
Vidas and Marblot ^FDA/HC^	2 (2)	51.5–67.5^ǂ^	99.0–99.2^ǂ^			
Vidas^FDA/HC^ or Wampole^FDA^ and Virablot^FDA^	1 (1)	57^ǂ^	99.5^ǂ^			
Vidas^FDA/HC^ or Wampole^FDA^ and Immunetics C6 Lyme ^FDA/HC^	1 (1)	68^ǂ^	99.5^ǂ^			
Immunetics C6 and Marblot ^FDA/HC^	1 (1)	50.6 (46.4, 55.7)	99.5 (99.1, 99.8)			
Cambridge^FDA^ and inhouse IB	1 (1)	50^ǂ^	100^ǂ^			
Undefined or non-commercial 2 tier tests	4 (5)	58 (41, 73)	96 (91, 98)			
Early (Stage 1) acute LD*	10 (19)	46.3% (39.1–53.7)	99.3% (98.3–99.7)	64.9	0.54	120.1 (51.9, 278.2)
Cambridge^FDA^ and inhouse IB	1 (1)	69.2^ǂ^	100^ǂ^			
Vidas^FDA/HC^ or Wampole^FDA^ and Marblot ^FDA^fd1a^/HC^hc1	2 (3)	32–41^ǂ^	99.5–100^ǂ^			
Vidas^FDA^fd1a^/HC^hc1 or Wampole^FDA^fd1a and Virablot^FDA^fd1a	2 (5)	34.4 (27.7, 41.6)	100.0 (97.5, 100.0)	816.8	0.67	1243.9 (21.9, 70.6k)
Vidas^FDA^fd1a^/HC^hc1 or Wampole^FDA^fd1a and Immunetics C6 Lyme ^FDA^fd1a^/HC^hc1	1 (1)	61^ǂ^	99.5^ǂ^			
Zeus ELISA ^FDA^fd1a^/HC^hc1 and Zeus AtheNA^FDA^fd1a	1 (1)	45.7^ǂ^	99.6^ǂ^			
Zeus ELISA and Marblot ^FDA^fd1a^/HC^hc1	1 (1)	39.2^ǂ^	99.6^ǂ^			
Immunetics C6 and Marblot ^FDA^fd1a^/HC^hc1	2 (2)	37.6–76.9^ǂ^	99.5–100^ǂ^			
Liason and Marblot ^FDA^fd1a^/HC^hc1	1 (1)	61.5^ǂ^	100^ǂ^			
Undefined or non-commercial 2 tier tests	4 (3)	60 (40,76)	96 (93, 97)			
Early (Stage 2) neurological and cardiac LD*	8 (4)	89.7% (78.3–95.4)	99.7% (98.4–99.9)	272.8	0.10	2629 (399, 17.3k)
Zeus ELISA and Marblot ^FDA^fd1a^/HC^hc1	1 (1)	83.3^ǂ^	95.6^ǂ^			
Vidas^FDA^fd1a^/HC^hc1 or Wampole^FDA^ and Marblot ^FDA^fd1a^/HC^hc1	1 (1)	80.0 (56.3, 94.3)^ǂ^	99.5 (99.1, 99.8)^ǂ^			
Vidas^FDA^fd1a^/HC^hc1 or Wampole^FDA^fd1a and Virablot^FDA^fd1a	1 (4)	63–96^ǂ^	100^ǂ^			
Immunetics C6 and Marblot ^FDA^fd1a^/HC^hc1	1 (1)	80.0 (56.3, 94.3)^ǂ^	99.5 (99.1.99.9)^ǂ^			
Unknown ELISA and Marblot ^FDA^fd1a^/HC^hc1	1 (1)	100^ǂ^	99^ǂ^			
Late (Stage 3) neurological and arthritis LD*	8 (18)	99.4%^H^ (95.7–99.9)	99.3% (98.5–99.7)	137.6	0.006	22.8k (3069, 169k)
Vidas^FDA^fd1a^/HC^hc1 or Wampole^FDA^fd1a and Marblot ^FDA^fd1a^/HC^hc1	1 (2)	90.1–100^ǂ^	99.5 (99.1.99.9)^ǂ^			
Vidas^FDA^fd1a^/HC^hc1 or Wampole^FDA^fd1a and Virablot^FDA^fd1a	2 (5)	99 (92, 100)	100 (95, 100)	1403.2	0.01	250k (1.8k, 33.9M)
Vidas^FDA^fd1a^/HC^hc1 or Wampole^FDA^fd1a and Immunetics C6 Lyme ^FDA^fd1a^/HC^hc1	1 (1)	100^ǂ^	99.5^ǂ^			
Immunetics C6 and Marblot ^FDA^fd1a^/HC^hc1	2 (3)	94.7–100^ǂ^	99.5 (99.1.99.9)^ǂ^			
Liason and Marblot ^FDA^fd1a^/HC^hc1	1 (1)	100^ǂ^	100^ǂ^			
Cambridge^FDA^fd1a and inhouse IB	1 (1)	43.9^ǂ^	100^ǂ^			
Zeus ELISA ^FDA^fd1a^/HC^hc1 and Zeus AtheNA^FDA^fd1a	1 (2)	100^ǂ^	95.6^ǂ^			
Zeus ELISA and Marblot ^FDA^fd1a^/HC^hc1	1 (1)	96.6^ǂ^	95.6^ǂ^			
Unknown ELISA and Marblot ^FDA^fd1a^/HC^hc1	2 (2)	100^ǂ^	95–99^ǂ^			
Convalescent (treated at stage 1) LD^+^*	7 (15)	58.2% (46.4, 69.2)	99.1% (97.8–99.6)	61.4	0.42	145.6 (56.1, 378.2)
Vidas^FDA^fd1a^/HC^hc1 or Wampole^FDA^fd1a and Marblot ^FDA^fd1a^/HC^hc1	1 (1)	26.7 (18.5, 36.2)^ǂ^	99.2 (97.6, 99.8)^ǂ^			
Vidas^FDA^fd1a^/HC^hc1 and Marblot ^FDA^fd1a^/HC^hc1	1 (2)	29–71^ǂ^	100^ǂ^			
Vidas^FDA^fd1a^/HC^hc1 or Wampole^FDA^fd1a and Virablot^FDA^fd1a	1 (4)	55–75^ǂ^	100^ǂ^			
Immunetics C6 and Marblot ^FDA^fd1a^/HC^hc1	2 (2)	25.7–57.9^ǂ^	97.9–99.5^ǂ^			
Liason and Marblot ^FDA^fd1a^/HC^hc1	1 (1)	68.4^ǂ^	98^ǂ^			
Zeus ELISA ^FDA^fd1a^/HC^hc1 and Zeus AtheNA^FDA^fd1a	1 (2)	22.2–68.3^ǂ^	95.6^ǂ^			
Zeus ELISA and Marblot ^FDA^fd1a^/HC^hc1	1 (2)	61.1–89^ǂ^	95.6^ǂ^			
Unknown ELISA and Marblot ^FDA^fd1a^/HC^hc1	2 (2)	64–75^ǂ^	99^ǂ^			
Convalescent (treated at stage 2 or 3) LD*	3 (6)	80.0%^H^ (70.8–86.8)	98.3% (96.6–99.2)	48.0	0.20	235.5 (129.7,427.8)
Vidas^FDA^fd1a^/HC^hc1 or Wampole^FDA^fd1a and Marblot ^FDA^fd1a^/HC^hc1	1 (1)	75 (53.3, 90.2)^ǂ^	99.2 (97.6, 99.8)^ǂ^			
Immunetics C6 and Marblot ^FDA^fd1a^/HC^hc1	2 (2)	70.8–80.5	97.9–99.5^ǂ^			
Liason and Marblot ^FDA^fd1a^/HC^hc1	1 (1)	75.6 ^ǂ^	98^ǂ^			
Zeus ELISA ^FDA^fd1a^/HC^hc1 and Zeus AtheNA^FDA^fd1a	1 (1)	100^ǂ^	95.6^ǂ^			
Zeus ELISA and Marblot ^FDA^fd1a^/HC^	1 (1)	81.3^ǂ^	95.6^ǂ^			

Sn estimate/ Sp estimate are from the meta-analysis bivariate model unless otherwise noted.

* Summary sensitivity and specificity across all tests at the specified stage of LD.

^ǂ^ Value or range of values for sensitivity and specificity as reported by the author.

Sn = sensitivity, Sp = specificity, DOR = diagnostic odds ratio. LR+ (positive likelihood ratio) and LR- (negative likelihood ratio) are based on the bivariate model and are different than direct calculations of LR+/LR- [48]. ELISA = enzyme-linked immunosorbent assay

^H^ Based on I^2^, a measure of between study heterogeneity, the heterogeneity in this group of studies was <60%, thus considered to be homogenous.

^FDA^ = Food and Drug Administration approved, ^HC^ = Health Canada approved, ^NC^ = non-commercial

Vidas = Vidas Lyme Screen, Wampole = Wampole Bb (IgG/IgM) ELISA test system, Marblot = MarDx Lyme Disease (IgG and IgM) Marblot Strip Test System, Virablot = ViraMed Biotech Borrelia B31 (IgG or IgM) Virablot, Immunetics C6 = Immunetics® C6 *B*. *burgdorferi* ELISA™, Cambridge = Cambridge, Human Lyme EIA for detection of antibodies, IB = immunoblot, Zeus ELISA = Zeus Lyme IgG or IgM ELISA Test system, Zeus AtheNa = Zeus AtheNA Muti-Lyte test system, Liason = Liason Borrelia IgG /IgM assay model 310870 (CLIA)

One study (1 line of data) was excluded from the analyses ([34]) because there was no specificity reported in the paper.

#### EIA vs. clinical diagnosis

First tier serological tests including enzyme-linked immunosorbent assays (ELISA) and other serological assays were evaluated in 23 studies (119 lines of data) with well-defined and whole cell targets, Table 3. There were a mix of FDA-licensed tests and in house tests. Similar to the two-tiered tests, test performance for patients with stage 1 LD was highly variable and had poor sensitivity. In later stages of LD, the sensitivity improved. The overall specificity varied by test and between studies more than was reported for the two-tier tests.

**Table 3:** Twenty three studies (119 lines of data) evaluating different assays (mainly 1^st^ tier tests) by stage of Lyme disease using hierarchical logistic regression models or simply sensitivity and specificity when less than four lines of data were available for meta-analysis.

Description	Studies (lines)	Sn estimate (95% CI)	Sp estimate (95% CI)	LR+	LR-	DOR (95%CI)
All stages*	11 (34)	82.0 (73.2, 88.4)	94.2 (90.0, 96.7)	14.2	0.19	74.2 (38.9, 141.5)
ELISA- C6 target	7 (11)	76.5 (60.0, 87.6)	97.1 (94.9, 98.4)	26.7	0.24	110.3 (44.6, 273.1)
Commercial ^FDA/HC^	4 (4)	91 (81, 100)	97 (94, 100)			
In house	3 (7)	64 (47, 80)	97 (95, 99)			
ELISA- VIsE target In house	1 (4)	63 (47,77)	98 (98, 99)	40.9	0.37	110 (66,183)
ELISA- pepC10 targetIn house	1 (1)	38.4 (32.7, 44)^ǂ^	99.0 (97.7, 99.5)^ǂ^			
ELISA- WCSCommercial ^FDA/HC^	3 (7)	70.6 (60.9, 78.8)	73.2 (59.5, 83.5)	2.63	0.40	6.57 (3.74, 11.6)
ELISA–fla and Osp targetsIn house	3 (5)	85.7 (54.8, 96.8)	91.2 (53.2, 98.9)	9.73	0.16	62.1 (8.2, 469.6)
LIPS–VIsE-OspC-V1sE In house	1 (1)	98 (93, 100)^ǂ^	100 (94, 100)^ǂ^			
IHA (B126 or B31) in house	1 (1)	100^ǂ^	95^ǂ^			
Early LD—stage 1*	15 (48)	54.0 (42.9, 64.8)	96.8 (95.0, 98.0)	17.1	0.47	35.9 (22.7, 56.9)
ELISA- C6 target	7 (11)	57.1 (46.7, 66.9)	97.5 (96.2, 98.5)	23.5	0.44	53.7 (23.8, 121.1)
Commercial ^FDA/HC^	3 (4)	65.6 (61.2, 69.7)	98.7 (98.3, 99.0)^¥^	48.9	0.35	140.3 (101.5, 193.9)
In house	3 (6)	48.4 (37.1, 59.8)	96.1 (93.5, 97.8)^¥^	12.6	0.54	23.4 (10.0, 54.7)
ELISA- WCS	6 (10)	77.5 (59.5, 89.0)	87.8, (73.9, 94.8)	6.35	0.26	24.7 (11.3, 60.6)
Commercial ^FDA/HC^	3 (6)	65.0 (47.3, 79.4)	94.5 (89.7, 97.3)	12.2	0.37	33.0 (14.9, 72.7)
In house	3 (4)	94.0 (54.0,100)	61.0 (53.0,69.0)	2.4	0.09	26 (2, 418)
Liason System Borellia Burgdorferi (diasorin)^FDA/HC^	1 (1)	64.4^ǂ^	98.0^ǂ^			
ELISA–Osp A-F targets in house	6 (22)	33.3 (19.3, 51.1)	97.5 (94.8, 98.9)	13.7	0.68	20.1 (10.8, 37.3)
PEG peptide–ELISA in house	1 (1)	100^ǂ^	100^ǂ^			
IHA (B126 or B31)in house	1 (2)	46–48^ǂ^	98–99^ǂ^			
BAT (B297 or 50772)in house	1 (1)	72^ǂ^	99^ǂ^			
Early LD–stage 2*in house	5 (6)	79.1 (66.1, 88.0)	97.7 (96.8, 98.4)	34.7	0.21	162.0 (66.1, 397.2)
ELISA- C6 target	3 (3)	80.5–100^ǂ^	95–97.9^ǂ^			
commercial ^FDA/HC^	1 (1)	80.5^ǂ^	97.9^ǂ^			
In house	2 (2)	95–100^ǂ^	95–96^ǂ^			
Liason System Borellia Burgdorferi (diasorin)^FDA/HC^	1 (1)	75.6^ǂ^	98.0^ǂ^			
ELISA–Osp A-F targetsin house	2 (2)	62–68^ǂ^	93–97^ǂ^			
Late LD–stage 3*	9 (20)	94.7 (86.0, 98.2)	96.1 (94.2, 97.4)	24.5	0.05	449.8 (120.0, 1686.3)
ELISA- C6 target	6 (10)	94.5 (79.4, 98.7)	97.5 (95.7, 98.6)	38.3	0.06	682.7 (97.1, 4801.2)
Commercial ^FDA/HC^	3 (4)	99 (95, 100)^¥^	99 (98, 99)^¥^	81.9	0.01	6553 (1593, 26964)
In house	3 (6)	89.3 (57.5, 98.1)^¥^	96.0 (93.1, 97.7)^¥^	22.5	0.11	202.0 (21.6, 1887.5)
ELISA- WCS Commercial ^FDA/HC^	2 (4)	93.3 (78.6, 98.1)	92.6 (86.3, 96.1)	12.5	0.07	172.5 (28.4, 1046.2)
ELISA–Osp targetsin house	3 (5)	84 (56, 96)	93 (90, 95)	11.9	0.17	70 (17, 286)
Liason System Borellia Burgdorferi (diasorin)^FDA/HC^	1 (1)	64.4^ǂ^	98.0^ǂ^			
Convalescent LD—stage 1*	5 (9)	77.8 (69.5, 84.3)	98.8 (98.4, 99.1)	63.5	0.23	282.3 (157.5, 506.0)
ELISA- C6 target	3 (5)	75 (61,85)	99 (98, 99)	61.5	0.25	242 (101, 583)
ELISA- WCSCommercial ^FDA^	1 (1)	81^ǂ^	98^ǂ^			
EIA- IFN-y targetIn house	1 (1)	67^ǂ^	96^ǂ^			
IHA (B126 or B31)in house	1 (2)	83–86^ǂ^	98–99^ǂ^			

Sn estimate/ Sp estimate are from the meta-analysis bivariate model.

* Summary sensitivity and specificity across all test at the specified stage of LD.

^ǂ^ Value or range of values for sensitivity and specificity as reported by the author.

^¥^ There was a significant difference between the commercial and in house test results.

Sn = sensitivity, Sp = specificity, DOR = diagnostic odds ratio. LR+ (positive likelihood ratio) and LR- (negative likelihood ratio) are based on the bivariate model and are different than direct calculations of LR+/LR- [48]. ELISA = enzyme-linked immunosorbent assay

^H^ Based on I^2^, a measure of between study heterogeneity, the heterogeneity in this group of studies was <60%, thus considered to be homogenous.

^FDA^ = Food and Drug Administration approved, ^HC^ = Health Canada approved, ^NC^ = non-commercial

IHA = indirect hemagglutination antibody test, ELISA = enzyme-linked immunosorbent assay, LIPS = luciferase immunoprecipitation systems, IFN-y = Interferon gamma, fla = flagellum, Osp = Outer surface protein.

ELISA performance on early stage 1 LD was investigated in 53 lines of data (16 studies), Table 3. These were further grouped by type of ELISA to understand where variation between studies was occurring. ELISAs targeting C6 included 4 lines (3 studies) on the Immunetics® C6 *B*. *burgdorferi* ELISA™ kit and seven lines (four studies) on unlicensed C6 ELISAs (Table 3). Accounting for whether the C6 ELISA was licensed explained 27% of the heterogeneity between studies and indicated the commercial ELISAs had an insignificant higher sensitivity 91(81–100) vs. 64(47–80) and similar specificity 97(94–100) vs. 97(95–99) over all stages of LD.

Whole cell sonicate (WCS) ELISAs for early LD included 10 lines from 6 studies Table 3. Three commercial test kits were included; Lyme Stat Test Kit, VIDAS Lyme Screen II and Wampole Bb ELISA test system (see S2 Text) across six lines and three studies. These performed differently than the four in house WCS ELISAs and the authors did not offer an explanation for the divergent results.

Recombinant proteins and/or chimeric proteins from Osp A-F (mainly A and C) targets were used to develop assays and tested on early LD patients. All studies were based on in house ELISAs with small sample sizes and the reported sensitivities varied from target to target ranging from 0–86%. Other assays included the use of Poly-ethylene Glycol (PEG)-peptide conjugates in an ELISA that reported 100% sensitivity and specificity on a small sample [49]. An indirect hemagglutination antibody (IHA) test using *B*. *burgdorferi* strains B31 and B126 had a low sensitivity 46–48% and a specificity of 98–99% which is comparable to other tests for early LD [50].

For assays used in cases of late LD, the sensitivity and specificity were higher and more consistent compared early LD results. A meta-regression controlling for test in the late LD category showed the Immunetics® C6 *B*. *burgdorferi* ELISA™ significantly out-performed (sensitivity and specificity p≤0.001) in house C6 ELISAs, the commercial WCS ELISAs (VIDAS Lyme Screen II and Wampole Bb (IgG/IgM) ELISA test system) and the in house ELISAs using various recombinant/ chimeric Osp targets, Table 3.

Not included in the meta-analyses in Table 3 was an evaluation of the reactivity of individuals previously vaccinated with the Osp A vaccine (removed from use in 2002); the results showed a 95% false positive rate with a WCS ELISA and a 5% false positive rate with a recombinant Osp A ELISA [51].

#### Immunoblots vs. clinical diagnosis

Across nine studies several commercial western blots were evaluated against clinical diagnosis of a range of LD. These included the Marblot test strip system by MarDx®, the Boston Biomedica Inc.(BBI) *B*. *burgdorferi* western blot test kit®, Immuno Dot Borrelia Dot Blot Test® and Viramed Biotech *B*. *burgdorferi* B31 Virablot®. Only one in house immunoblot was evaluated investigating the diagnostic sensitivity of a few recombinant targets.

The MarDx ® Lyme Disease Marblot Strip test system was evaluated in four studies (7 lines of data) on select LD groups and across early to late LD groups [52–55]. A meta-regression controlling for group indicated that the test performed significantly better on late LD patients, but whether the investigator evaluated results for IgM, IgG or both in parallel did not significantly affect the sensitivity or specificity.

The BBI western blot was evaluated in two separate studies using the same CDC test panel, but slightly different classification criteria; one used the BBI criteria (IgG required 3+ bands of 20,23,31,34,35,39 and 83 kDa and IgM 2+ bands 23,39,41, and 83 kDa) which has a different formulation for positive samples compared to the CDC criteria (IgG required 5+ bands 18, 23, 28, 30, 39, 41, 45, 58, 66, and 83 to 93 kDa and IgM 2+ bands 23, 39, and 41kDa) [35,46]. The results of the two criteria differed in sensitivity, which was 77% and 93% using CDC criteria respectively for IgM and IgG blots, compared to 93% and 100% using the BBI criteria for IgM and IgG, however the difference was not significant and specificity ranged from 77–99% with a gain in sensitivity resulting in slight losses to specificity, Table 4.

**Table 4:** Eight studies (33 lines of data) evaluating different immunoblots (2nd tier tests) at all stages of Lyme disease using hierarchical logistic regression models or the sensitivity and specificity data when less than four lines of data were available for meta-analysis.

Description*	Studies (lines)	Sn estimate (95%CI)	Sp estimate (95%CI)	LR+	LR-	DOR (95%CI)
**BBI Bb IgM or IgG western blot Test Kit^s^ (CDC criteria or BBI criteria)^FDA^**	2 (4)	91 (74, 97)	99 (82, 100)	115.8	0.09	1308 (29, 58491)
Immuno Dot Borrelia Dot Blot IgG/IgM Test^po^ (GenBio) ^FDA^	1 (4)	71 (58, 82)	95 (92, 97)	14.5	0.30	48 (22, 104)
MarDx Lyme Disease (IgG and IgM) Marblot Strip Test System^s^^,FDA/HC^	7 (20)	66.7 (54.6, 77.0)	93.5 (87.8, 96.6)	10.3	0.36	28.9 (12.2, 68.1)
Viramed Biotech Borrelia B31 IgG/IgM Virablot^s^^, FDA^	1 (2)	85 (65–96) ^ǂ^	77–90 ^ǂ^			

Sn estimate/ Sp estimate are from the meta-analysis bivariate model.

* IgM and IgG western blot tests conducted on early Lyme disease patients (<30 days) and only IgG tests conducted on later stages (>30 days) as per CDC guidelines.

^s^ subjective test interpretation: technician assesses banding pattern and line intensity. ^po^ partially objective test interpretation: technician assesses dot intensity.

^ǂ^ value or range of values for sensitivity and specificity as reported by the author.

Sn = sensitivity, Sp = specificity, DOR = diagnostic odds ratio. LR+ (positive likelihood ratio) and LR- (negative likelihood ratio) are based on the bivariate model and are different than direct calculations of LR+/LR- [48].

^H^ Based on I^2^, a measure of between study heterogeneity, the heterogeneity in this group of studies was <60%, thus considered to be homogenous.

^FDA^ = Food and Drug Administration approved, ^HC^ = Health Canada approved, ^NC^ = non-commercial

The Immunodot Borrelia Dot Blot IgG/IgM test by General Biometric Inc. was examined in one study; the results are shown in Table 4. An insignificant increasing trend in sensitivity with disease progression was noted (stage 1 50% (95%CI 19, 87), stage 2 70% (35, 93) and stage 3 100% (63, 100) [52]. Viramed Biotech *Borrellia burgdorferi* B31 IgG/IgM Virablot demonstrated a comparable sensitivity and specificity in one small study to the other immunoblots evaluated [35]. One in house recombinant immunoblot (data not shown) did not perform well in the published study with sensitivities ranging from 7 to 60 percent for different targets [56].

#### Tests for direct detection of *Borrelia burgdorferi* by bacterial isolation or PCR vs. clinical diagnosis

There are six studies, (13 lines of data) that examined bacterial isolation by culture and PCR detection of *B*. *burgdorferi* in a variety of human samples from cases of early and disseminated LD [57–62]. Meta-analysis was not possible within this group of studies because there were not enough lines of data within each detection method. The most commonly used medium is Barbour-Stoener-Kelly (BSK) medium, which has been modified by some authors to improve its sensitivity [63]. Three studies attempted to isolate *B*. *burgdorferi* from blood (serum/plasma) of patients with early LD (stage 1) and the sensitivity of this approach was 27%, 71% and 94% [57–59]. With respect to the latter sensitivity, it has been suggested that laboratory contamination may account for the very high sensitivity reported [64]. Two studies reported sensitivities of 62–81% from biopsy samples of EM during early LD [59,60], although both sample sizes were very small. Phillips et al. evaluated an “MPM” medium for detection of *B*. *burgdorferi* in the blood of LD patients that had been previously treated, but then relapsed [65]. They reported a sensitivity of 91.5% in these patients, however two studies were unable to reproduce these results and both demonstrated that the BSK-H culture was superior [66,67].

Three studies (eight lines of data) were captured with information on the use of PCR to identify *B*. *burgdorferi* in early LD [59,61,62]. Samples included blood and tissue biopsies and each PCR targeted different primers. Eshoo et al (2012) used blood samples and multi-loci PCR targeting eight different loci to both detect and genotype *B*. *burgdorferi*, the sensitivity was 62% (40–79) and the specificity was 100% [61]. Liveris et al (2012) used a nested PCR on serum samples and biopsy samples with a sensitivity of 40.6 and 42.6% respectively [59]. They also implemented a qPCR on plasma samples demonstrating a sensitivity of 33.8%. Two nested PCR primer sets targeting the Osp A gene were investigated in neurological LD, both acute and late cases using cerebral spinal fluid samples; they reported a sensitivity of 37.5–50% in acute cases and 12.5–25% in late cases [62]. Across the direct detection studies sensitivity was low and in most cases lower than the two-tier test regime, assays or immunoblots reported for early LD.

#### Inter-test comparisons

The results of inter-test comparisons are summarised in Tables 5–7. Note that in these tables we have positive agreement and negative agreement that indicate how well the two tests agreed to classify samples as positive or negative respectively. Thus, positive agreement is the probability that test 2 is positive if test 1 is positive and negative agreement is the probability that test 2 is negative if test 1 is negative. Table 5 has comparisons between the two-tier serological tests compared to other tests and Table 6 includes studies that examined various assays and immunoblots for agreement.

**Table 5:** Summary of agreement between tests reported in eight studies (10 lines of data) that examined two-tier serology testing compared to a different test or two-tier protocol.

Test 1- two-tiered	Test 2	Studies	PA estimate^a^	NA estimate^b^
Vidas Lyme Screen + MarDx Lyme Disease (IgG and IgM) Marblot Strip Test System ^FDA/HC^	kELISA: rVlsE1 ^NC^	1	82.0	68.1
	kELISA: C6 peptide ^NC^	1	81.0	63.7
fla_ELISA ^NC^ and MarDx Lyme Disease (IgG and IgM) Marblot Strip Test System ^FDA/HC^	ELISA (8 synthetic peptides)(IgG/IgM) ^NC^	1	82.8	66.7
	MarDx (IgG/IgM) ELISA ^**FDA/HC**^	1	89.7	58.3
unknown ELISA + MarDx Lyme Disease (IgG) Marblot Strip Test System ^FDA/HC^ 5/12 bands (includes bands 31kDa & 34 kDa)	two-tier: unknown ELISA + MarDx Lyme Disease (IgG) Marblot Strip Test System ^**FDA/HC**^ 5/10 bands- CDC criteria	1	92.0	100.0
IFA (unknown) + MRL diagnostics: Lyme Disease Bb genogroup 1 WB IgG or IgM^FDA^	two-tier: IFA (unknown) and BAT with Bb 297 vs 50772 (IgG/IgM) ^NC^	1	73.2	84.1
Wampole Bb (IgG/IgM) ELISA test system^FDA^ and MarDx Lyme Disease (IgG and IgM) Marblot Strip Test System^FDA/HC^	Immunetics® C6 *B*. *burgdorferi* ELISA™ (IgG/IgM) ^**FDA/HC**^	1^E^	98.5	49
(tests unknown, followed CDC guidelines)	Isothermal amplification/PCR/ESI-MS	1	57.1	28.6
(tests unknown, followed CDC guidelines)	PCR (CSF)—flagellin gene	1	5 (0, 25)	98.8 (93, 99)
Wampole Bb (IgG/IgM) ELISA test system ^FDA^ and MarDx Lyme Disease (IgG and IgM) Marblot Strip Test System^FDA/HC^	BSK culture (plasma >9ml)	1	19.2	n/a

^a^ PA = positive agreement estimate = On a sample of clinical LD patients, this is the probability of test 2 being positive if test 1 is positive.

^b^ NA = negative agreement estimate = On a sample of clinical LD patients, this is the probability of test 2 being negative if test 1 is negative.

^FDA^ = Food and Drug Administration approved, ^HC^ = Health Canada approved, ^NC^ = non-commercial

^NC^ = Not a commercial test, n/a = not applicable, ^E^ = early Lyme only (stage 1)

BSK = Barbour-Stoener-Kelly (BSK) medium, nPCR = nested polymerase chain reaction, qPCR = quantitative polymerase chain reaction

**Table 6:** Summary or range of agreement reported in 14 studies (51 lines of data) evaluating different tests paired against each other and tested on samples meeting the clinical definition of Lyme disease or a test panel.

Test 1	Test 2	Studies (lines)	PA estimate^a^	NA estimate^b^
EMIBA (Immune Complex)(IgG/IgM) ^NC^	MarDx Lyme disease EIA (IgM) test system ^**FDA/HC**^	1 (3)	66.1–77.8^P^	50-100^P^
	MarDx Lyme disease EIA (IgG) test system ^**FDA/HC**^	1 (2)	58.1–77.8^P^	50-100^P^
	CDC ELISA	1 (1)	100^P^	NA
	free antibody EMIBA ^NC^	1 (2)	98.4-100^P^	0-100^P^
	Immunowell Borrelia (Lyme) test^FDA^	1 (1)	56.3	50
	BION Borrelia Burgdorferi Antigen Substrate Slide^FDA^	1 (1)	81.3	25
	MarDx Lyme disease (IgM) Marblot strip test system ^**FDA/HC**^	1 (2)	55.6^P^-59.7	100
	MarDx Lyme disease (IgG) Marblot strip test system ^**FDA/HC**^	1 (2)	45.2–88.9^P^	100
	Immuno dot Borrelia dot blot M test^FDA^	1 (1)	62.5	75
	IgM Immunoblotting (2+ bands = 22,31,34,39,83) ^NC^	1 (1)	43.8	75
ELISA (8 synthetic peptides)(IgG/IgM) ^NC^	MarDx Lyme disease EIA (IgM & IgG) test system ^**FDA/HC**^	1 (2)	83.3–92.9^P^	61.5^P^-95.7
MarDx Lyme disease EIA (IgG) test system: PEG-IC ^NC^	MarDx Lyme disease EIA (IgG) test system ^**FDA/HC**^	1 (3)	33.0–81.1	61.5–100
MarDx Lyme disease EIA (IgM) test system: PEG-IC ^NC^	MarDx Lyme disease EIA (IgM) test system ^**FDA/HC**^	1 (3)	78.6–100	58.8–75.0
PEG peptide -ELISA (IgG/IgM) ^NC^	WCS ELISA -unknown ^**NC**^	1 (1)	77.4	n/a
kELISA: rVlsE1 ^NC^	kELISA: C6 peptide ^**NC**^	1 (1)	87	72.9
Human Lyme EIA for the detect of antibodies (Cambridge)^FDA^	CDC ELISA	1 (1)	96.1	100
Immunetics® C6 *B*. *burgdorferi* ELISA™ ^FDA/HC^	CLIA-VlsE assay (diasorin) ^**FDA/HC**^	1 (1)	70	99.1
IHA (B31 and B126 strain) ^NC^	Lyme Stat Test Kit^FDA^	1 (1)	80^P^	n/a
Immunowell Borrelia (Lyme) test^FDA^	BION Borrelia Burgdorferi Antigen Substrate Slide^FDA^	1 (1)^E^	90.9	33.3
Immuno dot Borrelia dot blot M test^FDA^	BION Borrelia Burgdorferi Antigen Substrate Slide^FDA^	1 (1)^E^	81.8	22.2
IgM Immunoblotting (2+ bands = 22,31,34,39,83) ^NC^	BION Borrelia Burgdorferi Antigen Substrate Slide^FDA^	1 (1)^E^	37.5	50.0
IgM Immunoblotting (2+ bands = 22,31,34,39,83) ^NC^	Immunowell Borrelia (Lyme) test^FDA^	1 (1)^E^	75.0	58.3
Immuno dot Borrelia dot blot M test^FDA^	Immunowell Borrelia (Lyme) test^FDA^	1 (1)^E^	81.8	77.8
Immuno dot Borrelia dot blot M test^FDA^	IgM Immunoblotting (2+ bands = 22,31,34,39,83) ^**NC**^	1 (1)^E^	63.6	88.9
RCBP ELISA chimeric proteins: A-93 (97) 1 B-C-Fla (64) (IgG/IgM) ^NC^	WCS ELISA -unknown	1 (3)^E^	100	82.6–100
OspC ELISA IgM ^NC^	Borreliacidal antibodies test (BAT) ^NC^	1 (1)^E^	80.9	100
IHA (B31 and B126 strain) ^NC^	Lyme Stat Test Kit^FDA^	1 (1)^E^	100	n/a
inhouse IB ^NC^	CDC IB	1 (1)	93.3	100
BBI research laboratories B. burdorferi IgM WB kit^FDA^	CDC WB IgM	1 (3)	90^P^-100^P^	0^P^-86.4^P^
BBI research laboratories B. burdorferi IgG WB kit ^FDA^	CDC WB IgG	1 (3)	74.3^P^-100^P^	0^P^-100^P^
Cambridge Biotech Human Lyme IgG western Blot^FDA^	CDC WB IgG	1 (1)	43.6 ^P^	100 ^P^
Cambridge Biotech Human Lyme IgM western Blot^FDA^	CDC WB IgM	1 (1)	64.3 ^P^	68.2 ^P^
MarDx Lyme disease (IgM) Marblot strip test system ^FDA/HC^	CDC WB IgM	1 (1)	78.9 ^P^	100 ^P^
MarDx Lyme disease (IgG) Marblot strip test system ^FDA/HC^	CDC WB IgG	1 (1)	47.0 ^P^	100 ^P^

^a^ PA = positive agreement estimate = On a sample of clinical Lyme disease patients, this is the probability of test 2 being positive if test 1 is positive.

^b^ NA = negative agreement estimate = On a sample of clinical LD patients, this is the probability of test 2 being negative if test 1 is negative.

^FDA^ = Food and Drug Administration approved, ^HC^ = Health Canada approved, n/a = not applicable

^NC^ = Not a commercial test, ^P^ = test panel used, ^E^ = early Lyme only (stage 1)

EMIBA = Enzyme-linked capture immune complex biotinylated-antigen assay

**Table 7:** Summary of agreement reported in four studies (six lines of data) evaluating culture and/or PCR of biopsy and various blood samples for the identification of *Borrelia* spp. in early stage1 Lyme disease patients.

Test 1	Test 2	Studies	PA estimate^a^	NA estimate^b^
BSK culture (plasma 3x 3ml)	BSK culture (serum 3x 3ml)	1^E^	100	75.9
BSK culture (whole blood 3ml)	BSK culture (serum 3x 3ml)	1^E^	33.3	82.1
BSK culture (plasma 3 x 3ml)	BSK culture–qPCR (plasma 3 x 3ml)	1^E^	100	54.3
BSK culture (biopsy 2mm)	qPCR (flaB) (biopsy 2mm)	1^E^	74.1	47.8
BSK culture (biopsy 2mm)	qPCR (recA) (biopsy 2mm)	1^E^	88.9	30.4
nPCR (flaB) (biopsy 2mm)	qPCR (recA) (biopsy 2mm)	1^E^	100	55.6

^a^ PA = positive agreement estimate = On a sample of clinical LD patients, this is the probability of test 2 being positive if test 1 is positive.

^b^ NA = negative agreement estimate = On a sample of clinical LD patients, this is the probability of test 2 being negative if test 1 is negative.

^E^ = early Lyme only (stage 1)

BSK = Barbour-Stoener-Kelly (BSK) medium, nPCR = nested polymerase chain reaction, qPCR = quantitative polymerase chain reaction

Table 7 contains studies that looked at various samples and culture sensitivity in early LD as well as the use of various PCRs to identify *B*. *burgdorferi* infection. In one study there was agreement between culture of serum vs. plasma, however whole blood classified more samples positive compared to serum resulting in little agreement [58,68]. The confirmation of *B*. *burgdorferi* presence in culture using qPCR both increased the sensitivity and shortened the length of culture time before a positive result could be obtained [69]. A study examining the sensitivity of direct qPCR targeting *flaB* or *recA* genes compared to culture of 2mm EM biopsy samples showed little agreement and qPCR targeting the *recA* gene was more sensitive compared to the fla B target [70].

### Diagnostic Test Performance in Early Lyme disease

Testing for LD in patients exhibiting signs and symptoms of LD for less than 30 days is challenging as the performance of available test protocols is not optimal for making clinical decisions. This is largely due to the time required for the infected individual’s immune system to mount a reaction. This is why researchers have explored the use of a variety of targets including VlsE and C6 expressed after infection, Osp C and Fla B expressed by the feeding tick to detect infection sooner [71,72]. However, cross-reactivity and genetic variability within the targets has limited the diagnostic performance of any single target [73,74]. Thus the results of expected sensitivities and specificities in Table 8 emphasize the importance of physician evaluation and informed judgement when deciding to treat rather than rely entirely on imperfect serological test protocols. Notable findings in the table include the higher specificity associated with the two-tier testing method and the poor and highly variable sensitivity of serological tests in the initial stages of disease when an individual is mounting an immune response to *B*. *burgdorferi*.

**Table 8:** Summary of the sensitivity and specificity of different testing options for early Lyme disease (stage 1) patients.

Description	Studies (lines)	Sn estimate	Sp estimate
Two-tier testing *	**10 (19)**	**46.3 (39.1–53.7)**	**99.3 (98.3–99.7)**
Cambridge^FDA^ and inhouse IB	1 (1)	69.2^ǂ^	100^ǂ^
Vidas^FDA/HC^ or Wampole^FDA^ and Marblot ^FDA/HC^	2 (3)	32–41^ǂ^	99.5–100^ǂ^
Vidas^FDA/HC^ or Wampole^FDA^ and Virablot^FDA^	2 (5)	34.4 (27.7, 41.6)	100.0 (97.5, 100.0)
Vidas^FDA/HC^ or Wampole^FDA^ and Immunetics C6 Lyme ^FDA/HC^	1 (1)	61^ǂ^	99.5^ǂ^
Zeus ELISA ^FDA/HC^ and Zeus AtheNA^FDA^	1 (1)	45.7^ǂ^	99.6^ǂ^
Zeus ELISA and Marblot ^FDA/HC^	1 (1)	39.2^ǂ^	99.6^ǂ^
Immunetics C6 and Marblot ^FDA/HC^	2 (2)	37.6–76.9^ǂ^	99.5–100^ǂ^
Liason and Marblot ^FDA/HC^	1 (1)	61.5^ǂ^	100^ǂ^
First tier EIAs*	**16 (48)**	**54.0 (42.9, 64.8)**	**96.8 (95.0, 98.0)**
ELISA- C6 target	7 (11)	57.1 (46.7, 66.9)	97.5 (96.2, 98.5)
Commercial ^FDA/HC^	3 (4)	65.6 (61.2, 69.7)	98.7 (98.3, 99.0)^¥^
In house	3 (6)	48.4 (37.1, 59.8)	96.1 (93.5, 97.8)^¥^
ELISA- WCS	6 (10)	77.5 (59.5, 89.0)	87.8, (73.9, 94.8)
Commercial ^FDA/HC^	3 (6)	65.0 (47.3, 79.4)	94.5 (89.7, 97.3)
In house	3 (4)	94.0 (54.0,100)	61.0 (53.0,69.0)
Liason System Borellia Burgdorferi (diasorin)^FDA/HC^	1 (1)	64.4^ǂ^	98.0^ǂ^
ELISA–Osp A-F targets in house	6 (22)	33.3 (19.3, 51.1)	97.5 (94.8, 98.9)
PEG peptide–ELISA in house	1 (1)	100^ǂ^	100^ǂ^
IHA (B126 or B31) in house	1 (2)	46–48^ǂ^	98–99^ǂ^
BAT (B297 or 50772) in house	1 (1)	72^ǂ^	99^ǂ^
Western blots (Marblot/ GenBio)*	**4 (8)**	**60.6 (42.7, 76.0)**	**96.8 (91.9, 98.7)**
**Direct Detection**			
Culture biopsies	2 (2)	61.8–80.8^ǂ^	NA
Culture blood	3 (3)	26.9–94^ǂ^	NA
PCR biopsies	1 (1)	42.6^ǂ^	NA
PCR blood (serum/plasma)	2 (3)	33.8–62^ǂ^	NA

Sn estimate/ Sp estimate are from the meta-analysis bivariate model unless otherwise noted.

* Summary sensitivity and specificity across all tests on early LD.

^ǂ^ Value or range of values for sensitivity and specificity as reported by the author.

Sn = sensitivity, Sp = specificity, DOR = diagnostic odds ratio. LR+ (positive likelihood ratio) and LR- (negative likelihood ratio) are based on the bivariate model and are different than direct calculations of LR+/LR- [48]. ELISA = enzyme-linked immunosorbent assay

^H^ Based on I^2^, a measure of between study heterogeneity, the heterogeneity in this group of studies was <60%, thus considered to be homogenous.

^FDA^ = Food and Drug Administration approved, ^HC^ = Health Canada approved, ^NC^ = non-commercial

Vidas = Vidas Lyme Screen, Wampole = Wampole Bb (IgG/IgM) ELISA test system, Marblot = MarDx Lyme Disease (IgG and IgM) Marblot Strip Test System, Virablot = ViraMed Biotech Borrelia B31 (IgG or IgM) Virablot, Immunetics C6 = Immunetics® C6 *B*. *burgdorferi* ELISA™, Cambridge = Cambridge, Human Lyme EIA for detection of antibodies, IB = immunoblot, Zeus ELISA = Zeus Lyme IgG or IgM ELISA Test system, Zeus AtheNa = Zeus AtheNA Muti-Lyte test system, Liason = Liason Borrelia IgG /IgM assay model 310870 (CLIA)

IHA = indirect hemagglutination antibody test, Osp = Outer surface protein

One study (1 line of data) was excluded from the analyses ([34]) because there was no specificity reported in the paper.

## Discussion

The 48 studies included in this analysis were all conducted in the United States from 1995 onwards. The samples included patients or historical samples where the clinical presentation fit the diagnosis of LD. Within the results we summarized results for all stages of LD, separate stages 1–3 LD and convalescent stages 1–3 LD to facilitate an evaluation of trends, similarities and differences by test, stage of disease and treatment status. There were a few studies that differentiated acute samples <7 days and early Lyme samples 7–30 days, but not enough to analyse predictive values within early LD. Similarly there were studies that used culture positive patients exclusively, however the culture status of the patients did not significantly account for the heterogeneity. Stage 1, 2, and 3 convalescent LD groups were sampled in a number of studies and are summarized separately from samples drawn pre-treatment as it is known that there are differences in the immune response depending on the length of LD prior to treatment [5,6].

In the United States it was recently estimated that less than 12% of Lyme disease tests were for true infections [75]. The LD test results for patients who do not meet the clinical criteria can be used to rule out LD, but a positive test is likely to be a false positive. Thus, the over use of these assays to diagnose LD has been an on-going discussion and challenge for topic-specialists and physicians [76]. The literature summarised in this systematic review was based on research conducted from 1995 when the CDC adopted the recommendations for two-tier testing of LD acquired in North America. Their goal was to improve the specificity of LD testing by recommending the use of a sensitive EIA followed by a more specific western blot for positive and equivocal samples [23]. Most of the research on diagnostic tests in North America were based on serology, mainly antibody based assays detecting an immune response against *B*. *burgdorferi*. As of May 2015 there were 42 tests approved by the FDA for use in the United States and 22 approved by Health Canada Medical Devices Branch for use in Canada, however only a few of these tests were evaluated in the primary literature and all the literature published since 1995 was conducted in the United States (see S2 Text).

Recent studies examining inter-laboratory agreement and the sensitivity and specificity of various test protocols noted that the C6 ELISA alone and the two-tier approach has superior specificity compared to proposed replacements and the CDC-recommended western blot algorithm has equivalent or superior specificity over other proposed test algorithms [77]. The findings of this review are in agreement with other authors that sensitivity was highest for ELISAs targeting C6 and these showed less variability in test sensitivity compared to other tests and test protocols [77]. The C6 ELISAs, particularly the commercial assays, had promising sensitivity, specificity and agreement of results with two-tier protocols, which is likely why the Immunetics® C6 *B*. *burgdorferi* ELISA™ has become widely used in place of some WCS assays. Although we did not summarize results of inter-laboratory agreement studies in this systematic review, the requirement for technical expertise and subjectivity in result interpretation for many LD tests, particularly western blots, contributes to poor agreement between technicians, tests and/or laboratories [77].

Factors that affect the sensitivity and interpretation of the results include type of sample and stage of disease in addition to possible variations in the type, target and conduct of the diagnostic tests. In this systematic review all relevant studies examining the efficacy of serological tests used serum samples from patients. There were no studies that employed the use of synovial fluid or cerebrospinal fluid for diagnosis of LD with serological assays. However in the last few years a number of studies have emerged from Europe on assays designed for cerebrospinal fluid samples in the diagnosis of neuroborreliosis which is a more common clinical presentation in Europe [78–80].

Throughout our results there was a positive association between duration of infection/ stage of disease and sensitivity of serological LD tests [34,47,60]. Thus, recommendations include re-testing after 30 days if the initial serological test was done during the early (non-disseminated) stages of infection and employing IgM assays as well as IgG assays to detect early immune reactions [21,81]. Other sources of heterogeneity between studies may include whether the case sampling frame included only samples from culture positive LD patients. Similarly, the impact of type of sample, prospective vs. retrospective patients and sample libraries or serum panels for test performance was investigated wherever possible in the analysis. The control group samples in the captured studies ranged from groups of healthy individuals from endemic and non-endemic areas to controls with diseases known to cross-react with LD diagnostic assays. Despite this, most studies reported a consistently high specificity for LD regardless of the composition of the control group and where there were differences (Tables 2–4), these were not statistically significant in most cases.

There was a wide range of assays identified in this systematic review including those assays that employed whole-cell sonicates mainly from *B*. *burgdorferi* B31 or other North American isolates to recombinant proteins targeting antigens that are highly expressed *in vivo* e.g. VlsE. Some of the captured research indicates that the VlsE targets improve test performance [45,82]. Similarly the C6 peptide which is derived from the VlsE lipoprotein has shown equivalent or better sensitivity compared to the WCS ELISAs in this systematic review, improved specificity for patients with often cross-reactive diseases and may also be used to identify some species of *Borrelia* acquired in Europe [47,73,74,82–84]. Subjectivity and inconsistency of the criteria used to evaluate western blot results has been noted as a source of confusion for patients and physicians in the interpretation of diagnostic results [85,86]. In studies where the CDC western blot interpretation was paired with different criteria, some showed gains in sensitivity with alternate criteria, but this was usually accompanied by a reduced specificity below an acceptable level [46].

Direct detection of *B*. *burgdorferi* from LD patient samples continues to be a challenge. *B*. *burgdorferi* requires culture in a complex medium for 8 to 12 weeks before the culture is considered negative, which makes this approach unsuitable in a clinical setting. Recent studies have attempted to improve the utility of culture by changing the protocol, for example, use of a 60 ml of BSK in a closed tube, incubated at 32–33°C for 8–12 weeks [58,59]. Another study used 15 ml and 2 ml starter cultures, then at day six seeded a long term culture in a caliper jar with 15 ml of fresh BSK for up to 16 weeks at 34°C [57]. Variations that had positive effects on culture growth included adding serum, a reducing agent and rifampicin [57–59]. The use of PCR to confirm bacterial isolation improves the sensitivity compared to visual confirmation by staining with acridine-orange and using dark-field microscopy or fluorescent microscopy [69]. The specimen, stage of LD and the laboratory technician’s experience has an effect on the likelihood of obtaining a successful *B*. *burgdorferi* culture. In early LD a biopsy sample from an EM lesion taken within the first week of symptoms has the highest sensitivity, whereas early disseminated infections have a higher sensitivity if isolation is attempted on large volume plasma samples [59,69].

Bacterial isolation has had limited success with late manifestations of LD and with cerebrospinal fluid and synovial fluid samples [87,88]. Research continues to focus on improving the sensitivity and speed of culture. Recent papers claiming major breakthroughs for *B*. *burgdorferi* isolation have failed validation [57] or could not be replicated [65] by others [64,67]. PCR for detection of *B*. *burgdorferi* DNA in LD patient samples is affected by many of the same limitations as culture with the exception that results may be obtained faster and PCR may be more sensitive in samples with a low concentration of *B*. *burgdorferi*. The variability of methodologies, gene targets and primers from study to study continue to impact the interpretation of the PCR results [59,61,62]. Overall, the sensitivities of PCR studies conducted in North America were lower than those that employed a two-tiered serology diagnostic protocol [59,61,62]. Due to the above limitations, bacterial isolation and PCR are not routinely used as diagnostic tools in clinical practise, although bacterial isolation is considered the gold standard to confirm diagnosis.

From the peer-reviewed literature we identified validation data from only a small proportion of licensed assays and for a number of “in house” tests which are used by several laboratories across North America. The performance of “in house” tests cannot be validated or critiqued as the composition of the test is not always publically available or evaluated in the peer-reviewed literature, thus comparing their performance to licensed tests is less informative. In studies looking at the variable performance of diagnostic testing schemes across laboratories it has been demonstrated that deviations from recommended diagnostic schemes often lead to a decrease in specificity and discordant results with approved testing schemes [89]. Thus, the performance of these “in house” assays and some of the older commercial assays have not been evaluated against well characterised panels of serum from patients with the full spectrum of LD clinical symptoms, with appropriate numbers of healthy controls and patients with look-alike diseases [32].

Future work on diagnostic tests for LD includes continued improvement in the sensitivity of all tests, particularly for early LD samples and the ability to distinguish between active infection and previous infections. On-going work into new immunoassay techniques and combinations of antigen targets that may help inform disease stage will hopefully improve LD diagnostics in the future [60,79,90]. Development of point-of care tests that do not require highly specialized technical skills and subjective interpretation of the results would help address some of the criticisms of immunoblot techniques. This systematic review summarizes research in North America on the accuracy of diagnostic tests for LD conducted since 1995. The performance of the commercially available Immunetics® C6 *B*. *burgdorferi* ELISA™ shows the most promise as a possible standalone test or as part of a two-tiered test protocol; however it did not overcome the low sensitivity of LD diagnostic tests in patients with early LD. Addressing this shortcoming is a significant challenge to improving LD diagnostics.

## Supporting Information

S1 Dataset Datasets from the tables. (XLSX)

S1 Text Protocol for Evaluation of the sensitivity and specificity of diagnostic test regimes for diagnosis of Lyme disease in humans, a systematic review and meta-analysis of the evidence. (PDF)

S2 Text 48 Studies Included in the SR and List of licensed tests for Lyme disease as of May 2015. (PDF)

S3 Text PRISMA checklist. (PDF)
