# Supplementary material for: The Accuracy of Diagnostic Tests for Lyme Disease in Humans, A Systematic Review and Meta-Analysis of North American Research
Source: PLoS One. 2016 Dec 21;11(12):e0168613. doi: 10.1371/journal.pone.0168613 (PMC5176185; doi:10.1371/journal.pone.0168613)
Supplement: S1 Text — (PDF) [file pone.0168613.s002.pdf]

Title:

Evaluation of the sensitivity and specificity of diagnostic test regimes for diagnosis of Lyme disease in humans, a systematic review and meta-analysis of the evidence.

Authors:

Lisa Waddell, Judy Greig, Mariola Mascarhenas, Shannon Harding, Pascal Michel

Public Health Risk Sciences Division of the Laboratory for Foodborne Zoonosis,

Public Health Agency of Canada.

Acknowledgements: Dr. Ian Young for feedback on forms. The Lyme disease stakeholder committee for feedback on the objectives, inclusion and exclusion criteria and content of this project.

Contact: Lisa Waddell, Tel: 519-826-2347 or email: lisa.waddell@phac-aspc.gc.ca

Important Dates:

Evidence published up to September, 2013, which was the date the scoping review search was conducted.

No search update is planned at this time.

Protocol version 1, initiated November 2014 – finalized March 2015

Review March-June 2015.

Analysis July 2015

Contents

Title:...1

Authors:..1

Important Dates: 1

Background...3

Methods 4

Scoping Review – Identification of Relevant Studies 4
Table 1: Describe the Types of studies, geographic distribution, participants, tests, standards used to evaluate the test. 6
Study Definitions 7
Definitions of Borrellia nomenclature: 7
Lyme disease: 7
Stages of Lyme disease: 7
Samples 8
Tests 9
Two-Tier Methods (Clinical Reference Standard test): 9
Table 2: Diagnostic Tests that have been investigated for Lyme Disease. 9
Objectives of the SR: 15
Review methods: 15
Study inclusion criteria: 15
Management of the SR 19
Analysis Plan/Options 19
Relevance Verification- General Characterisation Tool 24
Risk of Bias and Quality Assessment Tool 27
Data Extraction Tool 30

Background

Lyme disease (LD) is the most common tick-borne infection in Canada and much of the United States (Telford 1997; Ogden 2009). It was first recognized in North America in 1975 in the towns of Lyme and Old Lyme Connecticut as a result of an investigation into 51 cases (39 children) with a similar form of arthritis (Steere 1977). However, the history of *Borrelia* appears to be at least 5,300-years-old as the bacterium was identified in the mummified remains of the Tyrolean Iceman discovered in 1991 in the Italian Alps (Keller 2012).

In North America Lyme disease is caused by ***Borrelia burgdorferi*** sensu stricto while ***B. afzelii***, ***B. garinii***, *B. burgdorferi*, *B. spielmanii* and *B. bavariensis* cause disease in Europe with a wider variety of symptoms than reported in North America; ***B garinii*** is predominant in Asia.

Black-legged ticks of the Ixodes family transmit the spirochete through their bite. *I. scapularis* is the main vector in northeastern and upper midwestern USA and Canada while *I. pacificus* is the major vector in western USA (Gray 1998; Nelder 2014). The primary vectors of LD in Europe and Asia are *I. ricinus* and *I. persulcatus* respectively. The principal hosts of Ixodes ticks in North America include rodents, small mammals, birds and white-tailed deer.

Since first recognized in 1975, LD cases have increased progressively as the tick vectors have expanded their geographic range from the New England states into Canada and across some northern U.S. states (Hamer et al. 2010; Ogden et al. 2009) aided by migratory birds and terrestrial hosts (Leighton et al. 2012). There is increasing evidence that climate change will result in a further expansion of the tick vector range in Canada, resulting in increased future risk of LD among Canadians (Brownstein et al., 2005; Ogden et al., 2006a).

In North America early symptoms of infection may include a rash (characteristically a bulls eye rash), fever, headache and lethargy. If untreated, the disease can progress to chronic symptoms including arthritis, numbness or tingling in hands and feet and memory issues. The diagnostic tests available to confirm Lyme disease in humans are not perfect and have variable sensitivity and specificity depending on the stage of infection {{2014 linday, L.R. 2014;}}. There have also been concerns raised about the use of non-validated tests and test protocols {{2014 linday, L.R. 2014; 2013 Nelson,C. 2014;}}. The goal of this systematic review is to summarize the global evidence on the sensitivity and specificity of diagnostic tests and test regimes at various stages of Lyme disease.

Currently in Canada and the U.S. a two-tiered serology protocol is an accepted and validated test for disseminated Lyme disease diagnosis {{2014 linday, L.R. 2014; 2013 Nelson,C. 2014;}}. This two-tiered test is typically an enzyme immunoassay (EIA) to detect IgM or IgG antibodies in serum against *Borrelia burgdorferi*. There are a number of commercial ELISA kits

available that use either whole cell preparation of *B. burgdorferi* and/or recombinant antigens. This variation in target is likely a source of some heterogeneity. If a sample is positive or not determined by EIA then a Western blot test with better specificity is used to detect antibodies in serum against Borellia and confirms whether sero-conversion from IgM to IgG has occurred. Lindsay et al. (2014) summarize some of the strengths and weaknesses of these tests {{2014 linday, L.R. 2014;}}.

Methods

Scoping Review – Identification of Relevant Studies

A scoping review was conducted by Greig et al (2015) to identify, classify and characterise the main features of the Lyme disease literature published up to September 2013.

The PICO scoping review question (The Cochrane Collaboration, 2011):

"What is the current state of scientific knowledge on surveillance methods, prevention and control strategies, risk factors, and societal attitudes and perceptions towards LD disease in humans and *Borrelia* spp. in tick vectors and vertebrate reservoirs?"

Several systematic reviews were prioritized from the scoping study including an evaluation of the performance of Lyme disease diagnostic tests / test regimes for humans. The full paper was used to confirm the paper's relevance to the Lyme disease issue and describe the purpose, study design, location of the study, *Borrelia* sp., host species investigated, and vector species investigated. We also collected information on the sampling dates, diagnostic tests used, what extractable data is available in the paper and what is not extractable.

The scoping review included an advisory group that helped define the scope, provided background information and validated the interpretation of the results.

Scoping review search strategy: A pretested search strategy, adapted to the specific requirements of each database, was implemented in the following bibliographic databases: BIOSIS (via web of knowledge), CAB abstracts, Scopus, PubMed, PsycINFO, APA PsycNet, Sociological Abstracts, and EconLit during September 2013. There was no limitation on year of publication. To achieve an effective balance of sensitivity and specificity for identification of potentially relevant citations, the search was pre-tested in Scopus. The search strategy consisted of a targeted combination of specific terms designed to address the research question:

(lyme OR borrelia)

AND

("host" OR sentinel OR landscaping OR "vector" OR "vectors" OR "monitor" OR "monitoring" OR surveillance OR reservoir OR reservoirs OR prevalence OR educate OR education OR barrier OR barriers OR intervene OR intervention OR incidence OR rate OR prevent OR prevention OR control OR risk OR risks OR attitude OR attitudes OR perception OR perceptions or detection)

The capacity of the electronic search to identify all relevant primary research was confirmed by hand-searching reference lists from two primary research papers (Connally et al. 2009; Beaujean 2013), Clinical Practice Guidelines by the Infectious Diseases Society of America (Wormser 2006), one systematic review (Mowbray et al. 2012), three narrative reviews (Nardelli et al. 2009; Eisen et al. 2012; Gray 1999) and four conference proceedings (Annual Meeting of the German Society for Hygiene and Microbiology Congress, Dresden, Germany, September 28th to October 1st, 2003; VII International Potsdam Symposium on Tick-borne Diseases (IPS-VII) 2003; XVIIth main conference of the Polish Society of Epidemiology and Infectious Diseases, Warsaw, September 14-16, 2006; 50th Anniversary of the Polish Society of Epidemiology and Infectious Diseases, Scientific Workshop "The Man Facing Infectious Diseases" Bydgoszcz , 13-15 September 2007). The final search algorithms are available in the scoping review supplementary material (Appendix 1).

A search for grey literature on the websites of government and research organizations worldwide was conducted in February 2014 to complement the electronic database search; this resulted in the addition of 102 articles to the review (the full list of articles is available as supplementary material, Appendix 2). Only the following grey literature sources were considered for inclusion in the review: formal government and research reports; journal news, commentary, or editorial articles; and theses and dissertations.

Results: Of 16,516 records screened for relevance, 1843 relevant articles were analysed and categorized as follows: surveillance methods 722 articles, diagnostic tests 660, risk factors 452, efficacy of mitigation strategies 153, public knowledge, attitudes, or risk perceptions in North America 172, and economic burden of Lyme disease and/or cost-benefit of potential prevention/control strategies 57 articles.

Of the 660 diagnostic test papers 492 focused on diagnosis of Lyme disease in humans. These papers moved to the systematic review for further assessment and data extraction. The following is a summary of characteristics of studies identified to have evaluated a diagnostic test for Lyme disease in humans. These data will be used to inform the data extraction form for this SR and to confirm consistency in data.

Table 1: Describe the Types of studies, geographic distribution, participants, tests, standards used to evaluate the test.

Criteria	Categories	Number of Studies
**Total Studies**		492
**Focused on diagnostic tests**	Yes	463
	No	29
**Publication Date**	1980-1984	4
	1984-1985	36
	1990-1994	122
	1995-1999	118
	2000-2004	90
	2005-2009	85
	After 2010	36
**Continent**	North America	215
	Europe	260
	Australasia	1
	Asia	13
	Central, South America	2
**Study Design**	"diagnostic test evaluation"	424
	Observational- case study	10
	Observational- case-control	5
	Observational- cross-sectional	33
	Observational- cohort	2
	Observational- prevalence	3
	Observational- ?	1
	Experimental- Controlled Trial	1
	Experimental- Challenge Trial	3
	Unknown	1
***Borrelia* spp. studied**	Burgdorferi	456
	Garinii	87
	Afzelii	83
	Spielmanii	3
	bavariensis	1
	Valaisiana	6
	bissetti	5
	Other*	42
	NA	19
**Extractable Data**	Yes	454
	No	38
**Diagnostic Tests Evaluated**	Culture	93
	PCR	136
	EIA or IFA	157
	ELISA	268
	Western blot	234
	PFGE	8
	Biopsy	6
	C6	18

Other+	173
NA	3

*Other Borrellia investigated in 42 studies: *B. andersonii, B. americana, B. parkeri , B. hermsii, B. turicatae, B. lonestari, B. anserina, B. coriaceae, B. turicatae, B. japonica, B. recurrentis, Borrelia burgdorferi sensu lato, Borrelia burgdorferi sensu stricto, Borrelia lusitaniae, Borrelia finlandensis* sp. *Nov*
+ Other included a mix of dark field microscopy, several commercial assays, other assays, immunoblots, SDS-PAGE, BAT tests, DNA sequencing.

Study Definitions

Definitions of Borrellia nomenclature:

- *Borrelia burgdorferi sensu lato,* is considered to include: at least 15 recognized genospecies: *B. afzelii, B. andersonii, B. bissettii, B. burgdorferi sensu stricto (s.s.), B. garinii, B. japonica, B. lusitaniae, B. sinica, B. spielmanii, B. tanukii, B. turdae, B. valaisiana, B. californiensis, B. carolinensis and B. americana* .
- *B. burgdorferi sensu stricto* is mainly in N. America and is likely synonymous with *B. burgdorferi* reference in the N. American literature.

Lyme disease:

Lyme disease is characterised as an acute inflammatory disease that is caused by a spirochete (*Borrelia burgdorferi*) transmitted by ticks (genus *Ixodes*). Initial symptoms include a spreading red annular erythematous skin lesion (bulls eye rash / erythema migran) in 60-80% of cases and by fatigue, fever, and chills. Diagnosed Lyme disease cases are usually successfully treated with several weeks of antibiotics. If left untreated the infection may become disseminated and manifests as joint pain, arthritis, and cardiac and neurological disorders.

Lyme disease is diagnosed based on symptoms, physical findings (e.g., rash), and the possibility of exposure to infected ticks; laboratory testing is helpful if used correctly and performed with validated methods. (CDC definition)

Stages of Lyme disease:

While none of the symptoms occur in all patients there are some general guidelines to making informed choices about Lyme disease testing in individuals in N. America. (CDC website)

- **Early localised stage** (<2 weeks): known tick bite in endemic area (note different tests for different geographical regions), bulls eye rash (erythema migrans) usually appears 3-30 days after tick bite and is not painful or itchy. Only 60% will be ELISA positive at this stage. Serological tests are likely to be negative at this point; any negative results should be repeated in 4 weeks. NAAT (Nucleic Acid Amplification Test) to identify

Borrelia DNA in a sample such as a biopsy from a tick bite site may identify Borrellia exposure before an immune reaction would be detected. – Not really used.

- **Late localised stage** (2-4 weeks): Symptoms, red- expanding rash (EM), fatigue, chills, fever, headache, muscle and joint aches and swollen lymph nodes. IgM reaction typically would reach detectable levels at this point. EIA tests should be for IgM and IgG. If serology tested and negative, repeat in 4 weeks.
- **Early disseminated stage** (days to weeks post tick bite): Initial period where the infection spreads to other parts of the body. Symptoms include: Facial palsy (loss of muscle tone on the face), severe headache and neck stiffness due to meningitis, pain and swelling in joints, shooting pains, heart palpitations and dizziness. Without treatment many of these symptoms will resolve, but there is a greater risk of further complications.
- **Late disseminated Stage** (months to years post tick bite): Approximately 60% of untreated infections may lead to prolonged malaise including: intermittent bouts of arthritis, severe joint pain and swelling. Up to 5% of untreated patients develop neurological symptoms including shooting pain, numbness or tingling in hands and feet and problems with short term memory. IgG reaction should be detectable and will remain detectable for months to years. EIA or other assays only need to target IgG at this point. 80-90% of EM positive patients will be ELISA positive.
- Post treatment Lyme syndrome: It is estimated that 10-20% of patients treated for Lyme infection still have symptoms that last months to years. These include: muscle and joint pain, cognitive defects, sleep disturbance, and fatigue. There is no evidence that this is due to a persistent Borrelia infection and is thought to be an autoimmune reaction, continuing antibiotic treatment doesn't improve this condition. The serological tests will not be able to differentiate a new Lyme infection from previous positivity.
- Chronic Lyme disease: has been used to describe patients that fit the symptoms of Lyme disease but no evidence of current or past infection with Borrelia has been detected. There has been a lot of variation in the use of this term and its use is not well supported. (*Infect Dis Clin N Am* 22:341-60, 2008, *New Engl J Med* 357:1422-30, 2008).

Samples

- **Serology samples** are typically blood serum or biopsy plasma. These would be the most common sample taken for diagnostic tests.
- **Synovial fluid** (joint involvement), **cerebrospinal fluid** (neurological symptoms) and serology + ECG (cardiac symptoms) are used to test for disseminated Lyme disease depending on symptoms. NAAT (Nucleic Acid Amplification Test) to identify Borrellia DNA in a sample such as cerebrospinal fluid is possible, but not really used as the concentration is often below detection limits of the PCR.

Tests

All standardized and approved tests for Lyme disease are based on serology and designed to detect an immune response to antigens of Borrelia burgdorferi sensu stricto particularly IgG and IgM.

Two-Tier Methods (Index test):

Canada/ USA (since 1995) approved diagnostic testing sequence: When clinical symptoms such as rash, fatigue, headache, joint pain and/or neurological symptoms of Lyme disease are present (>1 week after an EM has appeared) and there is likely tick exposure (geography-time and activity history) then use **two-tier serological testing** = EIA- typically an ELISA (positive or equivocal) → Western blot (WB). List of approved tests from FDA and HC in separate pdfs.

- **Patient criteria**: A patient must have symptoms of Lyme disease e.g. bulls eye rash, history of being in a positive geographic region and possible or self-reported tick exposure. If yes and infection started >2 weeks prior, test with two-tier method, repeat after 4 weeks if negative.
- **First tier** is an EIA (ELISA= current methods or IFA= 'old method') that is quite sensitive. This test must be positive or borderline to indicate a second tier test. These tests commonly use whole cell antigens grown in vitro; V1sE is an immunodominant antigen and a small target within that antigen C6 26 amino acid peptide (commercial name Immunetics) are also approved for commercial use.
- **Second tier**: standardized immunoblotting (Western blot OR blots striped with diagnostically important purified antigens) that is quite specific. IgG positive is positive, IgM positive is positive but only for early disease (post EM, 1-2wks, to <1month or up to 6 weeks). How to score immunoblots has been standardized (lyme book) in N. America
- **Test conclusion**: If the specimen is positive on both tests, the patient specimen is considered positive. This has an average specificity = 99% or higher at reference centers (specificity of chronic Lyme = 97-100% and acute Lyme = 80-100%). High Sensitivity has been reported with few values or estimates.

Table 2: Diagnostic Tests that have been investigated for Lyme Disease.

Test	Definition	Other Notes
Antibody Tests		
**ARTCA, anti-recombinant tick calreticulin antibody**	Diagnosis of a tick bite: is measured in ng/μl and used as a biomarker of tick bites.	Not for diagnosis of Lyme disease.
**EIA**<br>**Enzyme Immuno Assay**	Detects IgM or IgG using an ELISA or IFA and either a whole cell preparation of *B. burgdorferi* or a purified antigen, recombinant antigen	Many commercial kits<br>Objective test interpretation<br>-Many validated

	(OspC), or recombinant peptide (e.g. C6 V1sE)	
**ELISA**<br>**Enzyme-linked Immunosorbant Assay**	- C_6 is a 26-amino acid sequence within the Borrelia membrane protein VIsE the test is a type of ELISA but specific for Borrelia strains (variety of Borelia) that cause Lyme disease and does not cross react.<br>- V1sE immunodominant antigen also approved for use.<br>- Whole-cell antigens – lysate ~100%Sn after EM stage, >1week.	C6 and V1sE based assays may also have features of detecting Eurasian *Borrelia* sp.<br>ELISA estimates the magnitude of the IgG/IgM humoral antibody response to the antigens. Results are objective, quantitative and correlate with the antibody titre.<br>Zeus ELISA- currently being tested by Robin.<br>http://www.zeusscientific.com/products/technology-systems/elisa/
**ELFA**<br>**enzyme-linked fluorescent immunoassay**		Commercial product: VIDAS® Lyme panel
**EMIBA**<br>**enzyme-linked immunoglobulin M capture immune complex (IC) biotinylated antigen assay**	This assay treats serum to dissociate immune complexes (thought to be present when infection is active) prior to the assay. Studies have shown improved Sn/Sp.	
**IFA**<br>**immunofluorescence assay**	(older tests)<br>**immunofluorescence assay (IFA)** is a powerful technique that utilizes fluorescent-labeled antibodies to detect specific target antigens. An antibody is a protein complex produced by B cells that Initiates an immune response against a target antigen. In this case, a fluorophore-labeled primary antibody directed against the suspected antigen is used to detect the presence or absence of the organism.	Some commercial tests previously approved<br>These are less used as they require a skilled microbiologist and cannot be scored objectively. Target IgG or IgM antibodies. Sensitive.
**CLIA**<br>**chemiluminescent immunoassay**	Qualitative presumptive detection of IgG and IgM antibodies. Intended to be first tier in 2 tier test.	Commercial name: DiaSorin LIAISON®Borrelia Burgdorferi = Uses recombinant V1sE antigens- objective machine reader.
**Immunoblot Tests**		Separate the bacterial antigens spatially on a solid support so that the Sp and complexity of the antibody response is revealed. The evaluation of a result is subjective in that the interpreter is looking

		for the existence of certain "bands". Qualitative tests. Sp=92%.
**WB**<br>**Westernblot**<br>**(aka protein immunoblot)**	Detects antibodies in a sample by the separation and detection of proteins (antigens, recombinant antigens or recombinant peptides to Borrelia) of a certain length by electrophoresis.<br>Can differentiate IgM from IgG.<br>***Postive IgM****= three bands are present: 24 kDa (OspC) *, 39 kDa (BmpA), and 41 kDa (Fla).*<br>***Positive IgG****= five of the following 10 bands are present: 18 kDa, 21 kDa (OspC) *, 28 kDa, 30 kDa, 39 kDa (BmpA), 41 kDa (Fla), 45 kDa, 58 kDa (not GroEL), 66 kDa, and 93 kDa*	Many commercial kits<br>Antigens are species specific (different targets for different Borrellia)<br>Higher Sp than EIA.<br>Subjective test interpretation.<br>-Many validated<br>-BANDS NOT USED: 31kDa (OspA) and 34kDa (OspB) not consistently detected.<br>-In Canada these tests are only *Borrelia burgdorferi* strain B31 based (Canlyme… true?)
**Striped blots**	Bands are in predefined positions so calibration (subjectivity) is avoided and outcome can be read by machine. Uses proteins (antigens, recombinant antigens or recombinant peptides to Borrelia)	Commercial: Virablots (Viramed) uses purified antigens (FDA approved 2009)
**Dotblot**	A mixture containing the molecule to be detected is applied directly on a membrane as a dot, and then is spotted through circular templates directly onto the membrane or paper substrate Uses proteins (antigens, recombinant antigens or recombinant peptides to Borrelia.	(Striped blot is a subset of this).
**SDS-PAGE**<br>**SDS-polyacrylamide gel – electrophoresis**	immunoblot with antigen targets to *Borrelia burgdorferi* (strain B31 in Canada) from serum	
**Antigen Capture Assays**	???	- Not validated for urine
	**Complement fixation test-** detects antibody or antigen in serum.<br>.	

**Multiplex immunoassay-**	any assay that that simultaneously measures multiple analytes (dozen or more) in a single run/cycle. Likely antibody protein arrays in this project.	Commercial: **Multiplex microsphere assay** (aka AtheNA Multi-Lyte test system) on the Luminex diagnostic platform. Approved first tier test, uses defined peptides.
**LIPSs luciferase immunoprecipitation systems**		
**IP Immunoprecipitation**	Is the technique of precipitating a protein antigen out of solution using an antibody that specifically binds to that particular protein.	
**ACIF anticomplement indirect immunofluorescence assay**	Indirect immunofluorescence utilizes a two-step technique, in which a primary, unlabeled antibody binds to the target, after which a fluorophore-labeled second antibody (directed against the Fc portion of the primary antibody) is used to detect the first antibody. This technique is more complicated and time consuming than direct immunofluorescence (because it requires a second incubation period); however, it is more sensitive because more than one secondary antibody can bind to each primary antibody, which amplifies the fluorescence signal.	
**A surface plasmon resonance (SPR) sensor**	has been used for the direct detection of Lyme borreliosis specific antibodies in blood serum.	
**MAT microscopic agglutination test**	is a serologic test that measures the patients serum ability to agglutinate live spirochetes (agglutins usually appear after >5days of infection). Uses live organisms, thus only performed in reference labs.	

**IEM**<br>**Immune (sorbent) electron microscopy**	immunoprobes (usually an antibody to borrelia) is used to identify antigens in the sample.	
**Lymphocyte transformation tests**	Tests for active infection via t-cell activity<br><br>Tests for chronic infection via CD3-/CD57+ (NK-cells) levels, which decrease with chronic infection.	Commercial name: Borrelia Elispot® LTT test approved by FDA in 2011. Tests for active T-cells against Borrelia, is good to diagnose an active infection. Should test negative 6-8 weeks after infection has resolved. Sn 84% and Sp 82-100%. CD3-/CD57+ NK-cells: monitoring the levels of these cells indicates whether there is a chronic infection and changes in these cells indicate whether a therapy is working.<br>-Not validated
**LPA**<br>**Lymphocyte proliferation assay**	is a test used to measure the ability of lymphocytes to proliferate in response to various stimuli	
**Quantitative CD57 lymphocyte assays**		- Not validated
**Reverse Western blots**		- Not validated
**BA**<br>**borreliacidal-antibody test,**	Serum is incubated with live Borrellia and inhibition is evaluated by % non-motile spirochetes, pH change, and flow cytometry. (High Sp) cumbersome to perform.	
**Direct Detection Tests**		
**Culture**	**Culture:** the bacterium is fastidious and requires a very complex growth medium and up to 12 weeks to grow. The medium; *Barbour-Stoenner-Kelly (BSK)* medium, commercial versions *BSK-II, BSK-H, Kelly medium Preac-Mursic (MKP).* It contains over thirteen ingredients in a rabbit serum base. Optimal temperature 32°C in a microaerobic environment.	Not used much.<br>Low Sn due to low concentration of Borrellia in samples.<br>-Many validated<br>- serum culture not validated<br>-expensive
**PCR**<br>**Polymerase chain reaction**	NAAT- nucleic acid amplification test. Is primarily used in research.<br>Detection of Borrelia DNA in sample. 18-83% from different samples and urine is not a suitable sample. Types qualitative (conventional and nested) or quantitative (real-time and competitive)	Can be used to detect Borrellia from lesions or from cerebrospinal fluid with neurological Lyme cases; however both suffer from low Sn and are not recommended.<br>Commercial: Sequence detection system and Light cycler are commercially available rt-PCR.

**Southern blot**	Is a method used in molecular biology for detection of a specific DNA sequence in **DNA** samples.	
**cell sorting of cell wall-deficient or cystic forms of *B. burgdorferi***	?	- Not validated
**immunofluorescence staining**		- Not validated
**Dark-field microscopy**	Used to view spirochetes. Dark field microscopy utilizes a special condenser which directs light toward an object at an angle, rather than from the bottom. As a result, particles or cells are seen as light objects against a dark background.	
**Fluorescence microscopy**	Fluorescent microscopy after staining with flourochrome dye acridine orange or fluorescence-labeled antibody.	
**FFM**<br>**Focus floating microscopy**	was developed to detect *B burgdorferi* in tissue sections	
**DNA Sequencing**		
**PFGE**<br>**Pulsed field gel electrophoresis**	is a technique used for the separation of large DNA molecules by applying an electric field that periodically changes direction to a gel matrix.	
**MLST**<br>**Multilocus sequence typing**	Is a technique in molecular biology for the typing of multiple loci. The procedure characterizes isolates of microbial species using the DNA sequences of internal fragments of multiple housekeeping genes.	
**Two-Tiered Methods**		
**Virablot Two-tier method**	A whole cell ELISA and IgG virablots with a V1sE band for early disease. This means 1 band= early disease and 5+ of 11 bands for late disease.	Commercial name: Virablot two-tier methods. (Benefits: avoids false positives of IgM blots and you do not need to know when infection occurred.)

Objectives of the SR:

Evaluation of the sensitivity and specificity of diagnostic test regimes for diagnosis of Lyme disease in humans, a systematic review and meta-analysis of the evidence.

1. Compile a list of published Lyme disease **diagnostic tests** for humans – from scoping review.
2. Extract or calculate sensitivity and specificity information reported for all stages and types of disease and for all types of Borrellia. Individual or combined tests
3. Compare the appropriateness of the current 2-tier recommendations in Canada to the performance of other diagnostic tests both approved and not currently approved for testing.
4. Evaluate the cost-benefit of tests that appear to perform better than the standard two tier method approved for use.

Review methods:

- Studies will be confirmed relevant to this SR (see relevance confirmation tool). Those with insufficient data to extract or insufficient detail (ie conference abstracts) will be excluded from further evaluation and summary.
- Studies will be evaluated by the QUADAS-2 tool {{2017 Whiting,P. 2008; 2016 Whiting,P.F. 2011; 2015 Whiting,P.F. 2013;}} for risk of bias and other methodological quality domains to assess the extent to which the results of each study or group of studies can be believed. (see Risk of bias and quality assessment tool). Chpt 9, the Cochrane Diagnostic Test Accuracy Handbook (Deeks 2009) and the more recent QUADAS-2 tool (2011) which has been updated from what the Cochrane chapter was based on, was used to construct this tool. Judgements of “at risk of bias” or “concerns regarding applicability” are judged based on the whether 1 or more domains indicated “high” or “unclear” deficiencies. Extensions of the QUADAS tool included a domain for comparison tests.
- The data extraction form will extract pertinent outcome information so assessment of sensitivity and specificity can be calculated post-hoc where not directly reported in the paper. (see data extraction tool)

Study inclusion criteria:

Study Design

- We expect these to be mainly diagnostic test accuracy studies, which are observational in nature and defined below. They were classified as diagnostic test studies at classification. No restriction on study design will be made at this point. The data will be grouped according to test, population/ stage of disease and study design for analysis.

- Typical diagnostic test: patients receive the index test, one or more other tests (optional) and the clinical reference standard (gold standard- what is used to diagnose patients).

Diagnostic test accuracy studies are typically **cross-sectional studies.** At inclusion in the study all patients are usually known to have or not have the condition of interest and there is usually not a lot of uncertainty about the status of the included individuals.

- **Delayed cross-sectional** studies occur when verification of the index test result is based on information that will only be available in follow-up after inclusion in the study.
- **Cohort type accuracy studies / single gate studies** are still cross-sectional in design. These studies employ a single set of inclusion criteria e.g. enroll everyone that presents to a clinic with symptoms of Lyme disease.
- **Case-control accuracy studies/ two-gate studies** are still cross-sectional in design. These studies employ different criteria for those with and without the target condition (Lyme disease). E.g. It may mean that patients with Lyme disease and without Lyme disease, but with another condition, were recruited from the same sampling base e.g. a clinic/ hospital. These are prone to bias as often they only include patients with severe forms of the disease of interest instead of a logical spectrum that reflects the disease in the population (these should be identified in study appraisal and perhaps omitted or sensitivity analysis with and without them during analysis). The generalizability of these studies may prevent it from addressing the clinical question.

Comparisons of Tests

- **Head to head** design: this is the strongest comparison that directly evaluates the test against each other. They can be fully paired where all participants received all tests AND the clinical reference standard.
- **Randomized direct comparison:** study participants are randomly allocated to receive the index test or the comparator AND all participants received the clinical reference standard test. This is the best not fully paired design to avoid selection bias.
- **Indirect comparisons:** While this may not be a study design, it can happen in a review. Indirect comparisons are prone to selection bias. If possible the comparisons reported should be based on fully paired or randomized designs.

Observational study: Assignment of subjects into a treated group versus a control group is outside the control of the investigator.

- **Cross-sectional:** Examines the relationship of a risk factor and outcome (disease) at a point in time on representative samples of the target population.
- **Cohort study**: is a study in which individuals with differing exposures to a suspected risk factor are observed through time for occurrence of an outcome
- **Case-control study**: compares exposure to the risk factor in subjects who have an outcome (the 'cases') with subjects who do not have the outcome, but are otherwise similar (the 'controls') and drawn from the same sampling frame.
- **Prevalence survey:** Measurement of an outcome at a point in time but doesn't measure or investigate potential predictors
- **Longitudinal prevalence:** A study that measures outcome (prevalence and distribution of disease only) at multiple points in time on the same population.

Experimental study: Each subject is assigned to a treated group or a control group before the start of the treatment

- **Controlled trial:** an experimental study in which people are allocated to intervention/comparison groups and evaluated for outcomes. **Randomized (RCT)** if authors specifically indicate random allocation of treatment/control.
- **Controlled before-and-after (CBA) study:** A study in which observations are made before and after the implementation of an intervention, both in a group that receives the intervention and in a control group that does not.
- **Uncontrolled before-and-after study:** observations are made on a population before and after receiving an intervention.

Participants

Parameters to assess before testing:

1. Stage of disease: skin symptoms (bulls eye rash) or tick bite or (disseminated disease AND tick exposure – known bites, living in an endemic area etc.)
2. Travel history (different test for Europe vs. N. America) – date of symptom onset (Different test for early vs. late disease).
3. Antibiotic use history as this may decrease the sero-response and increase the risk of false negatives.
4. Other autoimmune conditions as there may be a risk of false positive for Lyme due to cross-reactivity.
5. Previously positive for Lyme disease, a new test will not differentiate between a new or old infection.

Characteristics to Capture:

1. Age, sex
2. Comorbidities (especially autoimmune conditions)
3. Spectrum of Lyme disease patients and symptoms expected in the general population?

Clinical Reference Standard

Lyme disease is diagnosed based on symptoms, physical findings (e.g., rash), and the possibility of exposure to infected ticks; laboratory testing is helpful if used correctly and performed with validated methods. (CDC definition)

- Technically the clinical reference standard should be a test that is almost 100% Sn and Sp. The imperfection in the reference test leads to ***verification bias*** which can either under-estimate or over-estimate the test's accuracy. However, there is no test like this available for Lyme disease and many compare a test to patients that fit the clinical symptoms of Lyme disease. The clinical evaluation will be the clinical reference standard for this SR.

Index Test

- The index test for this SR on Lyme disease in N. America is the two tier method approved for use in patients suspected of Lyme disease (meet clinical definition);

"EIA → positive or equivocal → WB". Variability will occur in the antigen targets used. (This is the most accepted, but there is debate about whether it is the best and most error free as this test is not perfect.)

- Thus, the main comparison we are interested in is the two-tiered test vs. others tests.
- The target condition is diagnosis of Lyme disease.
    - There are 4 subcategories/ stages of disease and if possible we should capture or note that there are results for different stages of disease within a paper. The tests have a different Sn/Sp at different stages of infection: <2 weeks since exposure (early localised), 2-6 weeks since exposure (late localised), >4 weeks since exposure (early disseminated) and months to years since exposure (late disseminated).
    - Patients being screened should be considered based on their exposure (geography + tick bite) history and clinical symptoms (bulls eye rash or other malaise) to possibly have Lyme disease.
    - Post-treatment Lyme syndrome should not be included.
- Chronic Lyme disease: is not a widely accepted condition. Essentially it has defined the patients with Lyme disease symptoms but no exposure to Borrelia has been confirmed.

Comparator Test

- This is any test being compared to the index test. From this we get a relative diagnostic test accuracy of other tests compared to the index test.
- It may be a new test or variation of the two tiered method compared to the index test or the clinical reference standard, which we have defined as the current accepted standard for diagnosis of Lyme disease in Canada and the United States (detailed above) and diagnosis of Lyme disease based on clinical presentation and history respectively.

Management of the SR

This systematic review will be managed in Distiller (evidence partners, 2014) and each form will be completed by two reviewers working independently. Conflicts will be resolved by consensus. Data will then be exported to MS excel and prepared for summarization and analysis in STATA 13.

Relevance confirmation will confirm that the study is relevant to this SR and the study design.

Assessment of the methodological quality will follow the QUADAS-2 tool. All questions and definitions may be found in the Risk of bias and quality assessment tool

Data Extraction will include defining the test attributes, the population / stage of disease studied, and all relevant data including sample size, number positives for each test, sensitivity and specificity, other available data like ROC curves etc can be found in in the data extraction tool.

Analysis Plan/Options

Studies will be summarized and grouped by test, test comparison, stage of disease, and age of the population, targeted *Borrellia* spp., and study design. Appropriate comparisons, sensitivity and specificities, and other descriptive summaries will be presented in tables and graphs as appropriate.

Hierarchical random effect meta-analysis will be used and if possible meta-regression will be used to explore reasons for heterogeneity in STATA 13. If there are not enough studies, then sub group analysis will be used to evaluate the impact of different study attributes on the effect estimates. Meta-analysis provides us with an estimate of diagnostic tests accuracy and the uncertainty and variability of the findings around this estimate. Meta-regression can statistically compare the accuracy of two or more different diagnostic tests and describes how test accuracy varies with different tests, thresholds and other study characteristics.

It will be important to ensure that the studies are similar enough, particularly in the participants recruited- changes or differences in patient selection criteria will alter the spectrum of disease and non-disease in the population, which can impact tests accuracy.

Ultimately diagnostic tests and testing protocols will be compared for their positive and negative predictive values and the differences or apparent equivalencies across different diagnostic tests will be evaluated. Tables will summarize 1) the number of studies/individuals for each analysis, 2) diagnostic test accuracy, 3) comparative accuracy, 3) results of any heterogeneity investigation, 5) results of sensitivity analysis (10.3.5).

Evaluating accuracy of a test: Average Sn/Sp and potential summary ROC curve for varying thresholds will be most appropriate. Where prioritized, particularly for the two-tiered method, an investigation into heterogeneity will be considered if there are enough studies to do so. Important population and tests protocol characteristics have been identified and are captured in the data-extraction form.

- Which tests?
- 2-tier method approved

Comparing two or more tests: Pairwise or multiple tests can be compared. Considerations for multiple test comparisons (statistical issue) and what studies to include in the comparisons are needed (should the comparison be restricted to only the studies that make a direct comparison either by testing all patients or a random sample of patients?).

- Which tests?
- All others relative to two-tier
- Evaluate variations in two-tier, particularly approved vs. not approved
-

DATA: Definition of a test positive for each test, if there are multiple thresholds then we need to capture that information. Direct and indirect comparisons will be presented and part of the sensitivity analysis respectively to aid in the information being presented to decision makers. Substantial differences will be thoroughly explored and discussed.

1) **Binary data**: positive vs. negative
2) **Ordinal:** ordered set of categories (5) from definitely positive to definitely negative.
3) **Continuous or count**: outcome reported on a continuous scale or as a count (concentration or number of features observed). These are often dichotomized by pre-defined thresholds.

- For meta-analysis the ordinal, continuous or count outcomes need to be dichotomised, which means a **threshold** “cut-off” needs to be established.

- **Diseased** and **non-diseased** is established by the clinical reference standard and everything else is compared to that.
- 2x2 table can be drawn;
  - Sensitivity: the probability that the index test result will be positive in a diseased case. Sn=P(T+|D+)= a/a+c. Also referred to as detection rate, true positive rate or true positive fraction.
  - Specificity: the probability that the index test result will be negative in a non-diseased case. Sp=P(T-|D-)= d/b+d. Also referred to as true negative rate or true negative fraction.
  - False positive rate or false positive fraction = 1-Sp or b/(b+d) is often used.
  - Youden's index = Sn + Sp -1 → there is no probabilistic interpretation, it is an index of test accuracy which gives equal weight to testing positive and negative. (values close to 1 = higher accuracy than those close to 0)
- Predictive Values – *clinical relevance*
  - Positive predictive value (PPV) = probability that a diseased case is test positive = P(D+|T+) = a/(a+b).
  - Negative predictive value (NPV) = probability that a non-diseased case is test negative= P(D-|T-)= d/(c+d).
- Likelihood ratios
  - Bayesian MA- likelihood ratios can be used to update a pre-test probability of disease using Bayes theorem. If a test is informative you will get a higher LR than the pre-test probability and if it is not informative you will get a lower LR than the pre-test prob.
  - Positive likelihood ratio (LR+) = how many times more likely positive test results were in the diseased compared to the non-diseased group = P(T+|D+)/P(T+|D-) = Sn (1-Sp) = (a/(a+c))/(b/(b+d)). (>1 is an informative test)
  - Negative likelihood ratio (LR-) = how many times less likely negative test results were in the diseased group vs. the non-diseased group. = P(T-|D+)/P(T-|D-) = (1-Sn)/Sp = (c/(a+c))/(d/(b+d)). (<1 is an informative test)
- Diagnostic Odds Ratio
  - DOR= diagnostic accuracy of the index test as a single number that describes what the odds of obtaining a test positive result in a diseased rather than non-diseased person. This single number of accuracy is nice for meta-analysis, but has little clinical relevance. DOR= LR+/LR-= (Sn x Sp)/(1-Sn)x(1-Sp) = (ad)/(bc).
- Positivity thresholds
  - Sn and Sp typically vary inversely to each other and the goal of any test is to maximise Sn and Sp + the goals of the test to effectively identify disease with the least number of false alarms.

- ROC curves
  - A **ROC** curve is the graph of Sn and Sp that are obtained by varying the positivity threshold. The plot = Sn vs. (1-Sp) = true positive rate vs. false positive rate.
  - **AUC**= area under the curve = for the comparison of tests on the basis of their ROC curves takes into consideration their accuracy across a range of thresholds and is aided by single summary statistics like AUC. For the AUC, 1= perfect test and 0.5 = uninformative test. This represents the average sensitivity for a test taken over all specificity values.
  - Other values: partial areas under the curve, optimal operating points (defined by certain criteria).
  - **ROC and DOR**. If a ROC is symmetrical, then every point on the ROC has the same DOR. When the ROC is asymmetric the DOR will change with threshold values and the DOR can be used to describe these changes. This relationship forms the bases of MA on diagnostic test accuracy.
  - **Q*** is the point on the ROC curve where it intersects the downward diagonal line. This is the point where Sn and Sp are equal. This value has little meaning in practise.
  - **SROC =** summary ROC graphs = displays the results of individual studies in the ROC space. The points from each study can be sized to relay an understanding of precision and it is possible to add confidence intervals, but this makes the plot crowded. – the Meta- analytic outcomes that can be added include the summary ROC from the MA and the summary Sn and Sp
  - **Linked ROC:** these are used in analyses of paired tests. Thus, each study and each test is plotted by ROC with a line connecting the two tests in each study. This helps understand the difference in test accuracy within the study.
- **Forest Plots**
  - **Coupled forest plots:** Where two forest plots are put onto on graph typically Sn and Sp. This allows you to see where heterogeneity exists, but not to understand if there is a threshold type relationship. Often summary statistics are not provided with this plot.

MODEL FITTING

- **Moses-Littenberg SROC curve** similar to a fixed effect meta-analysis model as it doesn't include estimates of diagnostic test accuracy. This has been superseded by hierarchical models that allow for random effects. This SROC curve is produced from study Sn/Sp estimates transformed onto the log scale (logit). (10.4)
  - **D** (ln DOR)= logit (Sn)-logit(1-Sp) vs.
  - **S**(proportion of positive tests~ test threshold)=logit(Sn) + logit(1-Sp)

- The linear regression model *D=α + β S + error* characterises how lnDOR varies with S. Estimates of α and β are then substituted to get Sn across a range of values and produce a SROC.

- **Hierarchical models-** bivariate model (Reitsma 2005) and the hierarchical SROC (HSROC) model (Rutter 2001). Alternative bivariate models have been proposed by Arends 2008 and Chappell 2009. Both have distributions at two levels. Lower level= 2x2 data and binomial distributions. Upper level- random study effects are assumed to account for the heterogeneity. These two models are equivalent when no covariates are fitted.
  - Outputs: summary ROC curve, summary operating point (Sn/Sp) with 95%CI and a 95% prediction interval (assuming the model is correct this is where the true Sn and Sp of a future study should lie.)
- **Test comparison:** include all studies or only direct comparisons (less biased/less power).
  - **Bivariate model** is good when the cut-points are consistent or when you are comparing "kits" that produce positives/negatives. Any interpretation is only good for the tests at the cut-point compared and cannot be extrapolated. If the proportion of studies that used both tests is high, then a paired analysis should be done.
  - **HSROC model** can be used when different cut-points have been used. Thus, this is based on using the SROC for evaluating the two tests. Thus the analyst can look at whether test type has an effect on the shape and position of the SROC curve.
- **Software:**
  - **OpenBUGS** can fit the Bivariate and HSROC models**.**
  - **STATA:** bivariate model via glamm or xtmelogit. Assume the random effects are normally distributed. Metandi fits the bivariate or HSROC models without covariates {{2018 Harbord,Roger M. 2009;}}

Relevance Verification- General Characterisation Tool

Question	Answers	Explanation
**Is this primary research investigating the accuracy of diagnostic tests for Lyme disease in humans published in English, French, or Spanish?**	☐ Yes, primary research<br>☐ No, Relevant primary research only in short abstract (not enough detail)<br>☐ No, describes the use and promise of a new test without evaluating it on Lyme disease suspect or known samples. [ ]<br><br>☐ No, other reason(s) for exclusion:<br>☐ Literature review<br>☐ Predictive model<br>☐ Relevant to screening tests (of the general population) for Lyme disease reactivity.<br>☐ Relevant to other aspects of Lyme disease but not diagnostic tests: ___<br>☐ Not relevant: ___<br><br>*If "no" is selected, submit form without proceeding further.*	**Diagnostic Tests for humans**<br>To confirm a disease based on suspicion from clinical symptoms.<br>a) Studies evaluating the Sn and Sp of a diagnostic test or testing protocol.<br>b) Studies comparing the accuracy of 2 or more diagnostic tests for Lyme disease in humans.<br><br>**Lyme disease** is caused by the bacterium *Borrelia* spp. and is transmitted to humans by tick vectors.<br><br>**Primary research**: a study where the authors collected and analyzed their own data – may use quantitative or qualitative methods or both to investigate the research question and report original results.<br><br>**Exclude:**<br>• Studies *that did not evaluate a diagnostic test for humans*<br>• Exclude studies that present results for a new test without proper evaluation against an accepted reference test or samples of known disease status.<br>• Studies with *no relevant outcomes*<br>• Studies that are not primary research or do not have enough detail to properly evaluate (e.g. abstract)

**Were any diagnostic test outcomes sufficiently reported for potential use in meta-analysis?**	☐ Yes<br>☐ No, there is no extractable data in this paper.<br>*If NO – submit form without proceeding further*	
**This paper is about:**	☐ Evaluating the accuracy of a **diagnostic test.**<br>☐ Comparing 2 or more **diagnostic tests** relative to each other.<br>☐ Implementing screening tests for Lyme disease on a population. (exclude)<br>☐ Other ___	**Diagnostic test** would imply that the individual being tested has symptoms of Lyme disease (criteria 1 for the current 2-tiered diagnosis regime)<br>This may include inter-lab evaluations on samples with known disease status.<br>Accuracy/ Comparison of tests can be accomplished if they were applied on a sample of individuals.<br>**Screening tests** are used on the general population to evaluate exposure Borrelia, participants are not necessarily experiencing symptoms of disease. Often these are investigating a risk factor e.g. occupation for Lyme exposure.<br>**Other** – explain if it doesn't fit with the above.
**The test(s) has been applied on what type of diseased population?**<br>**(only note the <u>target disease e.g Lyme disease</u> and not the control groups.)**	☐ Suspect Lyme cases (indicate stage) ___<br>☐ Samples of known Lyme disease status for the purpose of test evaluation.<br>☐ Chronic/relapsing lyme disease<br>☐ General population in an endemic area (indicate area) ___<br>☐ High/low risk groups (e.g. occupation) ___<br>☐ Other ___	Samples being tested should be either suspected Lyme disease patients OR evaluation samples of known disease status.<br><br>General population screening and screening based on a risk factor status is not the intended use of the Lyme disease diagnostic tests and the performance of screening tests is outside the scope of this SR.
**In what year was the study conducted / published?**	☐ Prior to 1995 **and** did not evaluate a two-tier screening method [ ]<br>☐ After 1995 **OR** evaluated the two-tier screening method[ ]	Enter year. 1995 was when the two-tier standard emerged, but if a paper prior to 1995 evaluated this standard, we would like to include it.
**If exclusion criteria were checked in Q 1-4 above, submit**		

**the form.**		
INFO BOX<br>**Prior to proceeding identify the type of diagnostic test study you have and the type of tests being evaluated.**	☐ You may have a diagnostic test accuracy study (with a clinical reference standard and another test)<br>☐ Or you may have a diagnostic comparison study where two or more tests are being compared to each other. (Index and comparison tests.	These have different meanings and you will need to know what the clinical reference standard is, index test is and comparison tests to answer the QA and DE questions.<br><br>**If the study moving forward is not one of these it should have been excluded above!**

Risk of Bias and Quality Assessment Tool

Only answer for sections applicable to this paper. I.e.: reference test and index test or index test and comparison test.

SR Question: Evaluation of the sensitivity and specificity of diagnostic test regimes for diagnosis of Lyme disease in humans, a systematic review and meta-analysis of the evidence.		
**Patients: People suspected of having Lyme disease based on symptoms, and possible exposure**		
**Index Test: This would be any variation of the two-tiered method or a test that is in competition with the two-tiered method for diagnosing Lyme disease.**		
**Reference Standard: For this SR the two-tiered method is the clinical reference standard, thus a patient with symptoms consistent with Lyme disease for >2weeks can be tested with an EIA and if positive, a WB.**		
**Comparison tests: Any test being evaluated against currently accepted methods to diagnose Lyme disease**		
**Question**	Answers	Explanation
**Indicate the study set-up briefly.**	[Text]	Briefly indicate how participants were tested, in what order etc. so the analyst can understand the study.
**Domain 1: Patient Selection**		
**Describe methods of patient selection**	[text]	Copy and paste from paper.
**Was a consecutive or random sample of patients enrolled?**	☐ Yes ______<br>☐ Unclear _____<br>☐ No _____	YES: The method of recruitment was consecutive or random samples were taken from a consecutive series.<br>UNCLEAR: not enough information available<br>NO: The groups included were recruited separately.<br>**Consecutive series**: enroll a consecutive series (most appropriate) of patients fulfilling certain criteria or random-retrospective sampling from a series of patients. (increased Sp, {{2015 Whiting 2013;}})
**Was a case-control design avoided?**	☐ Yes ______<br>☐ Unclear _____<br>☐ No _____	**Diagnostic case-control**: severe cases/ known disease (very positives) + healthy controls (very negatives) = overestimate diagnostic accuracy – empirical evidence {{2015 Whiting 2013;}}
**Did the study avoid inappropriate exclusions?**	☐ Yes ______<br>☐ Unclear _____<br>☐ No _____	Inappropriate exclusions occur when difficult cases are excluded – these are the individuals suspected but not confirmed to have the disease = increased Sn/Sp estimates (over estimates diagnostic test accuracy)<br>Conversely, excluding patients who obviously have the disease can lead to an underestimation of the diagnostic test accuracy.
**Risk of bias due to patient selection (based on last 4**	☐ Low ROB<br>☐ Unclear ROB	Low RoB: The characteristics of the spectrum of patients included are representative and the method of recruitment was consecutive or

**questions)**	☐ High ROB	random samples were taken from a consecutive series.<br>UNCLEAR ROB: not enough information available<br>High ROB: The sample is not representative and/or the groups included were recruited separately and/or design and exclusions may bias results.<br>(Sn most affected bias in both directions. {{2015 Whiting 2013;}})
**Applicability - Is there concern that the included patients do not match the review question?**	☐ Low concern ___<br>☐ Unclear concern ___<br>☐ High concern ___	Low concern = There was a spectrum of likely Lyme disease patients included<br>High concern= patients included differ from those targeted by the review as they only focused on a subset of Lyme disease cases. Subsets by severity, demographics, differential diagnoses and comorbidities are typical.
**Domain 2: Index Test (Two – Tier method)**		
**Were the index test results interpreted without knowledge of the results of the clinical reference standard?**	☐ Yes ___<br>☐ Unclear ___<br>☐ No ___	(clinical reference standard results blinded)<br>*Indicate statement of blinding (or lack of) from text.*<br>*Describe the clear order of tests, and blinding methods.*<br>*We are using the approved, 2-tier EIA → WB + likely exposure protocol, which is not 100% Sn, Sp.(disagreements may arise due to incorrect classification by either tests instead of JUST the index test)*<br>*Otherwise note any Sn/Sp or discussion of the clinical reference standard's accuracy as reported.*
**If a threshold was used, was it pre-specified?**	☐ Yes ___<br>☐ Unclear ___<br>☐ No ___<br>☐ NA	Yes, threshold given<br>Unclear, not discussed at all.<br>No, threshold doesn't appear to be pre-specified.<br>NA- no threshold for this index test.
**Risk of bias- Could the conduct or interpretations of the index test have introduced bias?**	☐ Low ROB<br>☐ Unclear ROB<br>☐ High ROB	Low ROB, blinding, established thresholds and objective interpretation of the test<br>Unclear ROB– one or more deficiencies noted.<br>High ROB- concerns of bias due to deficiencies.
**Applicability – Is there concern that the index test, its conduct or interpretation differs from the review question?**	☐ Low concern ___<br>☐ Unclear concern ___<br>☐ High concern ___	Variations in test technology, execution, or interpretation may affect estimates. Given we are interested in exploring the variations, it is most important to note if there is a test that would not be applicable to this review question.
**Domain 3: Clinical Reference Standard =Clinical diagnosis of Lyme disease based on 1) symptoms, 2) history, 3) geography.**		

**Is the clinical reference standard likely to classify the target condition correctly?**	☐ Yes ___<br>☐ Unclear _____<br>☐ No _____	(acceptable clinical reference standard)<br>*(Disease severity associated with Sn {{2015 Whiting 2013;}}*
**Risk of bias-** Is there undue increased risk of bias on the described physician evaluation (clinical reference standard = physical symptoms + exposure to infected ticks) of patients included in the study?**?**	☐ Low ROB<br>☐ Unclear ROB<br>☐ High ROB	**Low ROB-** standard approach to diagnosis was described<br>**Unclear ROB**– one or more deficiencies noted.<br>**High ROB**- Approach to diagnosis differs from what is described on page 7 of the protocol.
**Domain 5 - Comparison Tests**		
**Were the comparison tests interpreted without knowledge of the results of the index test?**	Yes ___<br>Unclear _____<br>No _____<br>NA – no comparison tests	(comparison results blinded)<br>*Indicate statement of blinding (or lack of) from text.*<br>*Describe the clear order of tests, and blinding methods.*
**If a threshold was used, was it pre-specified?**	☐ Yes ___<br>☐ Unclear ___<br>☐ No ___<br>☐ NA	Yes, threshold given<br>Unclear, not discussed at all.<br>No, threshold doesn't appear to be pre-specified.<br>NA- no threshold for this index test.
**Risk of bias- Could the conduct or interpretations of the comparison test(s) have introduced bias?**	☐ Low ROB<br>☐ Unclear ROB<br>☐ High ROB	Low ROB, blinding, established thresholds and objective interpretation of the test<br>Unclear ROB– one or more deficiencies noted.<br>High ROB- concerns of bias due to deficiencies.
**Applicability – Is there concern that the Comparison test(s), its conduct or interpretation differs from the review question?**	☐ Low concern ___<br>☐ Unclear concern ___<br>☐ High concern ___	Variations in test technology, execution, or interpretation may affect estimates. Given we are interested in exploring the variations, it is most important to note if there is a test that would not be applicable to this review question.
**Domain 4 – Flow and Timing**		
**Is the time period between clinical reference standard and index test appropriate to be reasonably sure that the target condition did not change between the two tests?**	Yes _____<br>Unclear ______<br>No ______<br>Not reported ____	(acceptable delay between tests)<br>***Yes****, tests were taken at the same time or within a reasonable time of each other or there is a justification why delay is appropriate. Conversely- the minimum follow-up period was appropriate (where follow-up was necessary.)*<br>***No,*** *the tests were not taken within a couple months of each other. "disease progression bias" or "recovery bias"*<br>***Unclear,*** *the tests were taken more than a week apart unless this separation was justified as appropriate.*<br>*Paste the time period and justification in the textbox.*
**Did all patients receive the**	Yes ___	(differential verification avoided)

**same clinical reference standard?**	Unclear ____<br>No ___	*Unclear = not reported.*<br>*No= differential verification bias (where the results of a test are used to determine if the "gold standard" or clinical reference tests is used.)will over estimate Sn/Sp.*
**For studies with multiple comparator tests, was the whole sample or a random selection of the sample used to define which patients were tested with particular tests?**	Yes, whole sample<br>Yes, random sample ______<br>Unclear ______<br>No, partial sample _____<br>N/A – no comparator tests.	(partial verification bias avoided)<br>***Whole sample*** *and* ***random sample*** *of participants avoids selection bias to the best extent.* ***Unclear*** *= not reported and doesn't appear to be whole sample.* ***No, partial sample****= no randomization was used and only a selection of patients were tested with the reference standard.*
**Were all participants included in the analysis?**	Yes ___<br>Unclear ____<br>No ___	Yes: all patients included in the analysis.<br>Unclear: some patients who enrolled were lost to follow-up and this was evaluated and likely has little impact of the results.<br>No: Some patients were not included in the analysis and this was not justified/ explained.
**Risk of bias- Could the flow or timing of the study execution introduced bias?**	☐ Low ROB<br>☐ Unclear ROB<br>☐ High ROB	Low ROB- timing, application of tests and complete analysis is satisfactory.<br>Unclear ROB– one or more deficiencies noted.<br>High ROB- there was inappropriate timing, the application of the tests was NOT acceptable or there are unexplained missing observations that may bias the results.
**Other Questions**		
**Was there any report of inappropriate variation of results by technician, laboratory or instruments?**	Technician ____<br>Laboratory ____<br>Instruments ___<br>No, Examined and acceptable<br>Not reported	*If technician, laboratory or instruments were examined for inter-rater reliability and found to be unacceptable, indicate in the appropriate category.*
**Was this study free of commercial funding or are we confident the results were not influenced by a commercial enterprise?**	Yes ____<br>Unclear ___<br>No ___	(suppression of negative results)<br>**Yes,** there is no indication of funding or affiliation with a commercial company.<br>**Unclear,** one or more author has questionable affiliations.<br>**No,** funded by a commercial enterprise.

Data Extraction Tool

**General**

<table>
<tr><th colspan="3">Characterisation</th></tr>
<tr><td>What is the article language?</td><td colspan="2">☐ English
☐ French
☐ Spanish</td></tr>
<tr><td>What is the study design?
(Check all that apply)</td><td>☐ Diagnostic test accuracy studies
  ☐ Cross-sectional studies
  ☐ Delayed cross-sectional.
  ☐ Cohort type accuracy studies / single gate studies.
  ☐ Case-control accuracy studies/ two-gate studies.
☐ Diagnostic test comparison studies
  ☐ Head to Head
  ☐ Randomized Direct Comparison
  ☐ Indirect comparisons
☐ Observational study:
  ☐ Cross-sectional
  ☐ Cohort
  ☐ Case-control
  ☐ Prevalence survey
  ☐ Longitudinal prevalence
  ☐ Other: ___
☐ Experimental study:
  ☐ Randomized controlled trial (RCT)
  ☐ Non-randomized controlled trial
  ☐ Controlled before-and-after study (CBA)
  ☐ Uncontrolled before-and-after study
  ☐ Challenge trial (ChT)
  ☐ Other: ___
☐ Other, please specify: ___</td><td>Report ONLY study design(s) relevant to the research question.
Diagnostic test accuracy studies:
• Cross-sectional at inclusion of the study the participants are known to have or not have the condition, and there is not a lot of uncertainty about these individuals.
• Delayed cross-sectional occurs when the verification of the index test result is based on information that will only be available in follow-up after inclusion in the study.
• Cohort type accuracy studies / single gate studies, cross-sectional studies that employ a single set of inclusion criteria e.g. enroll everyone that presents to a clinic with symptoms of Lyme disease.
• Case-control accuracy studies/ two-gate studies, cross-sectional, employ different criteria for those with and without the target condition (Lyme disease). E.g. It may mean that patients with Lyme disease and without Lyme disease, but with another condition, were recruited from the same sampling base e.g. a clinic/ hospital.
Diagnostic Test Comparison
• Head to head design: this is the strongest comparison that directly evaluates the test against each other. They can be fully paired where all participants received all tests AND the clinical reference standard.</td></tr>
</table>

		• **Randomized direct comparison**: study participants are randomly allocated to receive the index test or the comparator AND all participants received the clinical reference standard test. This is the best not fully paired design to avoid selection bias.<br>• **Indirect comparisons**: While this may not be a study design, it can happen in a review. Indirect comparisons are prone to selection bias. If possible the comparisons reported should be based on fully paired or randomized designs.<br>• See protocol for other study design definitions.
**Population demographics**		
**In what continent was the study conducted?**	☐ N. America<br>☐ Europe<br>☐ Asia<br>☐ Other	Drop down.
**In what country was the study conducted?**	☐ text	Textbox- as reported.
**What populations were sampled?**<br>***(in the text box paste details about the control populations, the next question you can put the same details in about the Lyme disease population: e.g. # and description/disease they have.)***	☐ Patients suspected of Lyme disease- Basis of Diagnosis was:<br>☐ Symptoms: erythema migrans (EM) presence<br>☐ Symptoms: other rash, fever etc. ___<br>☐ History of tick bites _____<br>☐ History of geographical exposure ___<br>☐ Clinical diagnosis of Lyme disease by medical professional (type). ___<br>☐ Other _____<br>☐ Not described<br>☐ General population sample ___<br>☐ Healthy people _____<br>☐ Diseased _____<br>☐ Other ___	Some studies will have pulled a sample from the general population an then determined disease status / test status. Others may have used enrolment criteria to enroll healthy people, diseased people with non-Lyme diseases and Lyme disease (or suspected) cases selected from the same sampling base (ie: the same hospital, clinic etc.)<br>*If the sampling bases differ significantly this should have been reflected in first QA question.*

**Where were the participants recruited from? (if different for cases and controls note which group belongs to each category.)**	☐ Library of known disease status samples<br>☐ Panel<br>☐ Hospital<br>☐ Clinic<br>☐ General population<br>☐ Other ___	**Library:** Samples may have originated from a well characterised collection.<br>**Panel:** This is a group of well-defined/sero evaluated samples that are used as the "gold standard" for which to evaluate a test against or test inter/intra-laboratory agreement. e.g. CDC, BBI, EUCALB all have sample sets they use.<br>**Hospital:** samples from patients admitted to hospital.<br>**Clinic:** includes doctor's office and outpatient clinics.<br>**General population:** includes sampling blood donor clinics and different groups in the population or random sampling from the whole population.
**What type(s)/stages of Lyme disease did the sample population have?**<br>***(note if there were any relevant inclusion/exclusion criteria)***	☐ Early localized Lyme disease<br>☐ Early disseminated Lyme disease (< 2 month)<br>☐ Late Lyme disease (>2 months)<br>☐ Disseminated Lyme disease<br>☐ Cardiac Lyme disease<br>☐ Neuro Lyme disease<br>☐ Lyme Arthritis<br>☐ Lyme ACA (Europe Only)<br>☐ Chronic/Relapsing lyme disease.<br>☐ Other ____	Early Lyme disease is anyone for whom it has been less than two months since the onset of symptoms and localized is rash, EM etc. vs. disseminated which includes headache, fever, fatigue etc. Late Lyme disease is anyone for which it has been more than one month since onset.<br>Other forms are "chronic" forms of Lyme disease.<br>Acrodermatitis chronica atrophicans (ACA) is the third or late stage of European Lyme borreliosis.[1, 2] This unusual progressive fibrosing skin process is caused by an ongoing active infection with *Borrelia afzelii*.<br>**Chronic/Relaping lyme disease**: these are individual diagnosed and treated for lyme disease for whom the symptoms and infection come back.
**What was the prevalence of Lyme disease in the population from which the sample was taken (pre-test probability)? (%)**	☐ Text<br>☐ NR	Indicate as reported.
**What was the total number of participants (observations) in this study?**	☐ Text	Sum all the participants from each group together.

**Describe the attributes of the studied population (as reported):**	☐ Male ___<br>☐ Female ___<br>☐ Age (as reported) ___<br>☐ Ethnicity/Race ___<br>☐ Occupation ___<br>☐ Other ___	Describe the attributes of the population studied as reported in the paper.
**Were co-morbidities investigated as risk factor for a positive test?**	☐ Yes, describe co-morbidities and if there were any associations reported.___<br>☐ No	
**Were there other risk factors that affect the test results reported in this paper**	☐ Yes ___<br>☐ No	Co-morbidities should be reported in the previous question. This could include age, sex, race, geography etc.
**Was antibiotic treatment an inclusion/exclusion criteria, or captured as a possible risk factor?**	☐ Yes, inclusion<br>☐ Yes, exclusion<br>☐ Yes, risk factor: describe proportion of sample that had treatment and whether that was reported to influence the results. ___	Inclusion = all participants had antibiotic treatment<br>Exclusion = None had antibiotic treatment<br>Risk Factor = the effect of treatment was examined in the study. Please report evaluation.
**Tests evaluated**		
**What is the clinical reference standard test?**	☐ Describe _______<br>☐ N/A	The ***clinical reference standard test*** for this SR is a patient with likely Lyme infection exposure/ symptoms that warranted testing for Lyme disease.
**What is the index test(s)?**<br>*In the text box put a short name/description of the test that you will use in data extraction: e.g. ELISA in house IgG or Virotech WB IgG & IgM.*	☐ 2-tier method, check tests off and provide details above AND describe test order and decision logic here:_____<br>☐ Enzyme Immuno Assay (EIA)____<br>☐ immunofluorescence assay (IFA) ____<br>☐ ELISA____<br>☐ EMIBA (enzyme-linked immunoglobulin M capture immune complex (IC) biotinylated antigen assay) ___<br>☐ chemiluminescent immunoassay(CLIA)___<br>☐ immunochromatographic assay ____<br>☐ Western blot (WB)____<br>☐ Striped blot (Virablots)___<br>☐ Dotblot ____<br>☐ Lymphocyte proliferation	The ***Index test*** for this SR is the use of a two-teir method (EIA→ WB in series) to confirm the diagnosis of Lyme disease in patients presenting with symptoms synonymous with Lyme Disease (def at bottom of test table in protocol). Indicate if tests were "approved" by a governing body. (FDA approved list pdf)

	assay (LPA)___<br>☐ borreliacidal-antibody test (BAT) ___<br>☐ Complement fixation test<br>☐ SDS-PAGE ____<br>☐ luciferase immunoprecipitation systems (LIPSs)___<br>☐ Immunoprecipitation<br>☐ anticomplement indirect immunofluorescence assay (ACIF)___<br>☐ Immune(sorbent) electron microscopy (IEM)___<br>☐ microscopic agglutination test (MAT)____<br>☐ Multiplex immunoassay ___<br>☐ Multiplex microsphere assay<br>☐ surface plasmon resonance (SPR) sensor<br>☐ Other assay ____<br>☐ Culture _____<br>☐ PCR<br>☐ Conventional PCR__<br>☐ Nested PCR___<br>☐ Real time PCR__<br>☐ competitive PCR___<br>☐ Southern blot ___<br>☐ Dark-field microscopy _____<br>☐ Culture confirmation<br>☐ Fluorescence microscopy ___<br>☐ Culture confirmation<br>☐ Focus floating microscopy __<br>☐ Pulse Field Gel Electrophoresis ____<br>☐ Other molecular/typing test___<br>☐ Other______	
**Description of Comparison tests:**	☐ Comparison Test 1: commercial name<br>☐ Comparison Test 2: commercial name<br>☐ Comparison Test 3: commercial name<br>☐ Comparison Test 4: commercial name	Check off the number of comparator tests in the study AND provide their commercial name in the textbox if applicable. (a set of questions will appear for each comparison test.)

**What are the comparator test(s)?**	☐ Enzyme Immuno Assay (EIA)____<br>☐ immunofluorescence assay (IFA) ____<br>☐ ELISA____<br>☐ EMIBA (enzyme-linked immunoglobulin M capture immune complex (IC) biotinylated antigen assay) ___<br>☐ chemiluminescent immunoassay(CLIA)___<br>☐ immunochromatographic assay ____<br>☐ Western blot (WB)____<br>☐ Striped blot (Virablots)___<br>☐ Dotblot ____<br>☐ Lymphocyte proliferation assay ___<br>☐ borreliacidal-antibody test (BAT) ___<br>☐ Complement fixation test<br>☐ SDS-PAGE ____<br>☐ luciferase immunoprecipitation systems (LIPSs)___<br>☐ Immunoprecipitation<br>☐ anticomplement indirect immunofluorescence assay (ACIF)___<br>☐ Immune(sorbent) electron microscopy (IEM)___<br>☐ microscopic agglutination test (MAT)____<br>☐ Multiplex immunoassay ___<br>☐ Multiplex microsphere assay<br>☐ surface plasmon resonance (SPR) sensor<br>☐ Other assay ____<br>☐ Culture _____<br>☐ PCR<br>☐ Conventional PCR__<br>☐ Nested PCR___<br>☐ Real time PCR__<br>☐ competitive PCR___<br>☐ Southern blot ___<br>☐ Dark-field microscopy _____<br>☐ Culture confirmation<br>☐ Fluorescence microscopy ___<br>☐ Culture confirmation<br>☐ Focus floating microscopy __	*A comparator test is a test being compared to the index test or the CRS test. There may be more than one listed here. This occurs in a situation where the* clinical *reference test has not been used for comparison.*<br>See test table page 8 for a description of each test.

	☐ Culture confirmation<br>☐ Pulse Field Gel Electrophoresis ____<br>☐ Other molecular/typing test___<br>☐ Other______<br>☐ **Multi-tier method**, check tests off and provide details above AND describe test order and decision logic here (how does this differ from the standard 2-tier method?): ______	
**Description of the Test**	☐ Attributes for the following (index, comparison 1,2, 3, or 4) test ____<br>☐ Commercial Name (if reported) ___	The following Questions will be inserted after the index test and 4x after the comparator test. They will be hidden unless the questions about index/comparison test (above) are answered.
**What were the targets for the test? (specify test/IgG or IgM in text box)**	☐ **Purified Antibody _____**<br>☐ **Purified Antigen ____**<br>☐ **Whole-cell preparation ____**<br>☐ **Recombinant antigen (specify IgG or IgM in text box)**<br>☐ **14kDa ___**<br>☐ **P17 (Osp17) ___**<br>☐ **18kDa ___**<br>☐ **21kDa (OspC) ___**<br>☐ **22kDa (OspC)**<br>☐ **23kDa (OspC)**<br>☐ **28kDa**<br>☐ **30kDa**<br>☐ **35kDa**<br>☐ **39kDa (BmpA)**<br>☐ **P39**<br>☐ **45kDa**<br>☐ **58kDa**<br>☐ **66kDa**<br>☐ **83kDa**<br>☐ **93kDa**<br>☐ **P83/100**<br>☐ **BBK32**<br>☐ **DbpA**<br>☐ **DbpB**<br>☐ **41kDa (fla)**<br>☐ **flaA**<br>☐ **flagellin 41-i**<br>☐ **flagellin P41-G**	Specify the antibody, antigen, whole cell preparation used.<br>**RECOMBINANT /Purified ANTIGENS-** *specify IgG or IgM* **flagellin P41-G or 41-I, FlaA, BBK32, P39, P35, OspA, OspB, OspC, OspE, OspF, V1sE and DbpA**<br>**OspC** induces a potent early immune response and is also one of the most diverse proteins in the *Borrelia* proteome. Yet, at least 70% of the amino acid sequence is conserved among all 21 known OspC types<br>*Ameican criteria: immunoblots IgM = 1 band OspC, 39kDa or 41kDa. IgG 5 of 10 bands = 93, 66, 58, 45, 39, 30, 28, 21, or 18kDa.*<br>**RECOMBINANT PEPTIDES** *specify IgG or IgM*<br>**C6 (IR6) peptides- (**Immunetics®C6 B. burgdorferi) when EM was present Sn 66.5% vs. 35% without an EM and Sp=98.9%<br>**pepC10-** recombinant OspC protein, less hetero than OspC,

	☐ OspA ___<br>☐ OspB ___<br>☐ OspE ___<br>☐ OspF ___<br>☐ V1sE___<br>☐ Other _____<br><br>☐ **Recombinant peptide (specify IgG or IgM in text box)**<br>☐ C6 /IR6<br>☐ pepC10<br>☐ other _____<br>☐ **PCR**<br>☐ Primer target(e.g. a repeat region) ___<br>☐ Primers e.g. [p1, p2] ___<br>☐ primer size (base pairs)___<br>☐ **microscopy**<br>☐ dye/stain ___<br>☐ target description ____<br>☐ **Other ______**	not great Sn/Sp.
**What type of Borrelia was used to develop the target for the test? (e.g. B. burgdorferi B31 type strain)**	☐ {Text}	
**What sample(s) is used?**	☐ Serum<br>☐ Whole blood<br>☐ Joint fluid<br>☐ Cerebral spinal fluid<br>☐ Other: _____	
**Was the sample preabsorbed to decrease crossreactivity of the test? (describe in textbox)**	☐ Text	*If the sample was preabsorbed to decrease crossreactivity, please describe the preabsorption in the textbox. (leave blank if not reported.)*
**Optical Density (OD)/ Wavelength (λ)**	☐ text	*As reported, the optical density /the wavelength used in the test. (leave blank if not reported.)*
**Dilution factor (1:300)**	☐ text	*As reported, what was the dilution factor for this test? If multiple report all. (leave blank if not reported.)*
**Time to get results (days)**	☐ text	*How long does it take to test the sample and get a result? Please report in DAYS ;)*
**Reported Sensitivity (%)**	☐ text	*As reported for this test-sample combination, what was the sensitivity of this test? (leave blank if not reported.)*

**Reported Specificity (%)**	☐ text	*As reported for this test-sample combination, what was the specificity of this test? (leave blank if not reported.)*
**Is the output reading from this test objective or subjective?**	☐ objective<br>☐ subjective	*Objective= read by a calibrated machine or definitive line to indicate a positive test.*<br>*Subjective= requires a skilled technician to interpret the test readings e.g. most western blots.*
**What threshold was used to categorise samples**	☐ Negative ___<br>☐ Positive___<br>☐ Other ___	For meta-analysis the ordinal, continuous or count outcomes need to be dichotomised, which means a **threshold** "cut-off" needs to be established.<br>***Other*** *would be to define the unequivocal samples.*
**Is this test FDA approved?**	☐ Yes<br>☐ No	*See FDA approved pdf.*
**Is this test Health Canada approved?**	☐ Yes<br>☐ No	*See HC approved pdf.*
**Test cost ($)**	☐ Text	*Please indicate if reported. If $ is not available, qualitative information (e.g. expensive) is also welcome.*
**Was the description of the test and methods described in sufficient detail to answer the above questions?**	☐ Yes<br>☐ No, referenced to another paper that will have to be procured.<br>☐ Not described or referenced.	*The main purpose of this question is to flag studies that will require follow-up. ie: another paper needs to be procured.*
**If two different samples were used, were they sampled at appropriate times for the test?**	☐ Yes<br>☐ No ___	• EM for direct identification = acute phase of disease<br>• Serology = 4+ weeks after exposure<br>• Others: disseminated disease doesn't matter.
**Were there reasons for discordant test results across laboratories?**	☐ Yes, describe ___<br>☐ Not reported<br>☐ NA	• There have been many papers evaluating consistency of testing across labs; indicate hypotheses made by the author for why results were discordant.<br>• Discordant results were noted, but no reasons for differences explored.<br>• NA, not applicable.
**Were cost comparisons described in the paper?**	☐ What cost comparisons were reported for the tests in this paper? ___	• We are interested in numerical and qualitative discussion about the cost

	☐ Is there a cost-benefit description for the test(s)? (ie, new test can diagnose earlier and leads to reduced treatment costs) ___	of tests in option 1.<br>• Option 2 is asking if there is any quantitative or qualitative discussion about the cost-benefit of one test vs. the other. This can include an evaluation of early diagnosis leading to more effective treatments, reductions in healthcare costs for cases of Lyme disease etc.
**DATA ANALYSIS**		
**What relevant outcomes are not in an extractable format (i.e. graphs)?**	☐ Outcomes NOT sufficiently reported are: ___<br>☐ ROC graphs<br>☐ sROC graphs<br>☐ other graphs ___	Only answer this question with respect to **test performance** (e.g. test validity/reliability measures) and not for other outcomes that may be reported.
**Data Collection Form:**		**You will need a new data collection form to report each set of outcomes.**
**Was any test performance outcomes sufficiently reported for potential use in meta-analysis?**	☐ Yes<br>☐ No, there is no extractable data in this paper.<br>*If NO – submit form without proceeding further*	
**Indicate the characteristics of this line of data:**	☐ Clinical Referent Standard vs. index test<br>☐ Clinical Referent Standard vs. comparison test<br>☐ Index test vs. comparison test<br>☐ Referent test ___<br>☐ Referent threshold (or positive criteria)___<br>☐ Index test ___<br>☐ Index test threshold(or positive criteria) ___<br>☐ Comparison test ___<br>☐ Comparison test threshold (or positive criteria)___<br>☐ Sample type ___<br>☐ What stage of Lyme disease? ___<br>☐ Other descriptors ___	**See definitions for tests in protocol.**<br>Describe the tests, sample and any other pertinent information about this line of data. Only fill in what is needed.<br>The test names here just help to link it to the descriptions earlier in the form. Include the thresholds for each test in this line of data where applicable.<br><br>**Stage of Lyme disease:** early acute, late acute, early disseminated, late disseminated, chronic. See page 7 for details.

**Dichotomous/ Ordinal Data**	☐ Raw 2x2 table data:<br>☐ D+|T- ___<br>☐ D+|T+ ___<br>☐ D-|T- ___<br>☐ D-|T+ ___<br>☐ Define disease (D+/-) ___<br>☐ Define test (T+/-) ___<br>☐ Significance test? [text]<br>☐ P-value [text]<br>☐ If greater than two groups (ie, ordinal data with non-dichotomized categories), specify data for other groups ___<br>For other tests use a new form if more appropriate	**Only answer based on how outcome data are REPORTED**<br><br>**Dichotomous:** Sufficient information includes:<br>• Numerator ***and*** denominator, ***or***<br>• proportion + EITHER numerator or denominator ***or***<br>☐ Measure of association (e.g. odds ratio, relative risk) + EITHER a measure of variability (SE, CIs, variance) *or* an exact P-value<br><br>**Raw 2x2 table:**<br>**D+**
**Diagnostic test outcomes**	☐ Sensitivity: ___<br>☐ Define Sn (ie Referent test vs. index test etc.) ___<br>☐ SE ___<br>☐ Variance ___<br>☐ Lower CI ___<br>☐ Higher CI ___<br>☐ Specificity: ___<br>☐ Define Sp (ie Referent test vs. index test etc.) ___<br>☐ SE ___<br>☐ Variance ___<br>☐ Lower CI ___<br>☐ Higher CI ___<br>☐ PPV, positive predictive value ___<br>☐ NPV, negative predictive value ___<br>☐ False positive rate ___<br>☐ Youden's index ___<br>☐ Positive likelihood ratio ___<br>☐ Negative likelihood ratio ___<br>☐ Diagnostic Odds Ratio ___<br>☐ Area Under (ROC) Curve ___<br>☐ Q* ___<br>☐ Other ___	**Sensitivity/Specificity of a test:** define what the Sn refers to (tests compared + other important information)<br>**Sensitivity:** the probability that the index test result will be positive in a diseased case. aka detection rate, true positive rate or true positive fraction.<br>**Specificity:** the probability that the index test result will be negative in a non-diseased case. aka true negative rate or true negative fraction.<br>False positive rate or false positive fraction = 1-Sp<br>**Youden's index** = Sn + Sp -1. It is an index of test accuracy<br>Positive predictive value **(PPV)** = probability that a diseased case is test positive = P(D+|T+)<br>Negative predictive value **(NPV)** = probability that a non-diseased case is test negative= P(D-|T-)

		**Positive likelihood ratio** (LR+) = how many times more likely positive test results were in the diseased compared to the non-diseased group (>1 is an informative test)<br>**Negative likelihood ratio** (LR-) = how many times less likely negative test results were in the diseased group vs. the non-diseased group. (<1 is an informative test)<br>**Diagnostic Odds Ratio, DOR**= diagnostic accuracy of the index test as a single number that describes what the odds of obtaining a test positive result in a diseased rather than non-diseased person. DOR= LR+/LR-= (Sn x Sp)/(1-Sn)x(1-Sp)
**Raw continuous index or comparison test outcome data (Disease positive vs. Disease negative group based on Referent standard or index test): Raw continuous data in each group (final outcome measure)**	☐ Counts in group 1 ___<br>☐ SD in group 1 ___<br>☐ N in group 1 ___<br>☐ Counts in group 2 ___<br>☐ SD in group 2 ___<br>☐ N in group 2 ___<br>☐ Define group 1 ___<br>☐ Define group 2 ___<br>☐ P-value (exact only) ___<br>☐ T value ___<br>☐ For matched studies, specify pre/post correlation ___<br>☐ Outcome units ___<br>☐ Outcome scales (i.e. lowest/highest possible values) [Detection limit or analytical sensitivity] ___<br>☐ Threshold for dichotomization as suggested by the author.	**Continuous:** Sufficient information includes:<br>• Mean, sample size, + EITHER a measure of variability (e.g. SD, CIs) *or* exact P-value/t-value ***or***<br>• Sample size and P-value/t-value from t-test ***or***<br>• Difference in means and a measure of variability (SD, SE, CIs, variance) ***or***<br>• Difference in means, sample size, + EITHER a common SD *or* an exact P-value /t-value<br><br>For meta-analysis the ordinal, continuous or count outcomes need to be dichotomised, which means a **threshold** "cut-off" needs to be established for positive / negative groups.
**Difference in means from index or comparison test outcomes (between Disease positive and disease negative**	☐ Difference in means (value) ___<br>☐ N (total sample size) ___<br>☐ Common SD ___<br>☐ SE ___	

**groups as determined by the reference standard test or index test)**	☐ Variance ___<br>☐ Lower CI ___<br>☐ Higher CI ___<br>☐ P value (exact only) ___<br>☐ T value ___<br>☐ Outcome units ___<br>☐ Outcome scale (i.e. lowest/highest possible value) [Detection limit or analytical sensitivity] ___<br>☐ Other ___
**Measure of Association**	☐ Computed effect size/measure of association:<br>☐ Measure of association (value) ___<br>☐ Specify measure (OR, RR, etc.) ___<br>☐ N in group 1 ___<br>☐ N in group 2 ___<br>☐ Define group 1 ___<br>☐ Define group 2 ___<br>☐ SE ___<br>☐ Variance ___<br>☐ Lower CI ___<br>☐ Higher CI ___<br>☐ Was outcome adjusted for other variables? Please specify: ___
**Was there any relevant statistical analysis on test performance outcomes? (Please check all that apply and indicate the test outcome(s))**	☐ t-test (specify type e.g. paired, pooled) ___<br>☐ McNemar's exact test ___<br>☐ Fisher's exact test ___<br>☐ Chi Square ___<br>☐ Pearson's rank correlation ___<br>☐ Mann-Whitney U Test ___<br>☐ Kendall's tau b test ___<br>☐ Wilcoxon Rank Sum Test ___<br>☐ ANOVA ___<br>☐ Kruskal-Wallis One-Way ANOVA by Ranks ___<br>☐ Linear Regression Analysis ___<br>☐ logistic regression model ___<br>☐ mixed effect model ___<br>☐ GEE (Generalized Estimating Equations) ___<br>☐ GLMMs (Generalized linear mixed models) ___<br>☐ MCMC (Markov chain Monte

	Carlo estimation) ___<br>☐ Other ___<br>☐ No statistical analysis performed	
**37)** Were there any OTHER relevant outcomes that have not been extracted?	☐ ___	Although we aim to extract all data. When data is presented as a summary of the sample and broken into small subsets e.g. culture positive and negative Lyme patients or EM+ and EM- subsets. It may not be worth extracting all the subsets at this point until we know that we can use the subset information. Please note in this box the subset data that is available for which tests.
**38) Additional comments:**	☐ ___	

Organising results:

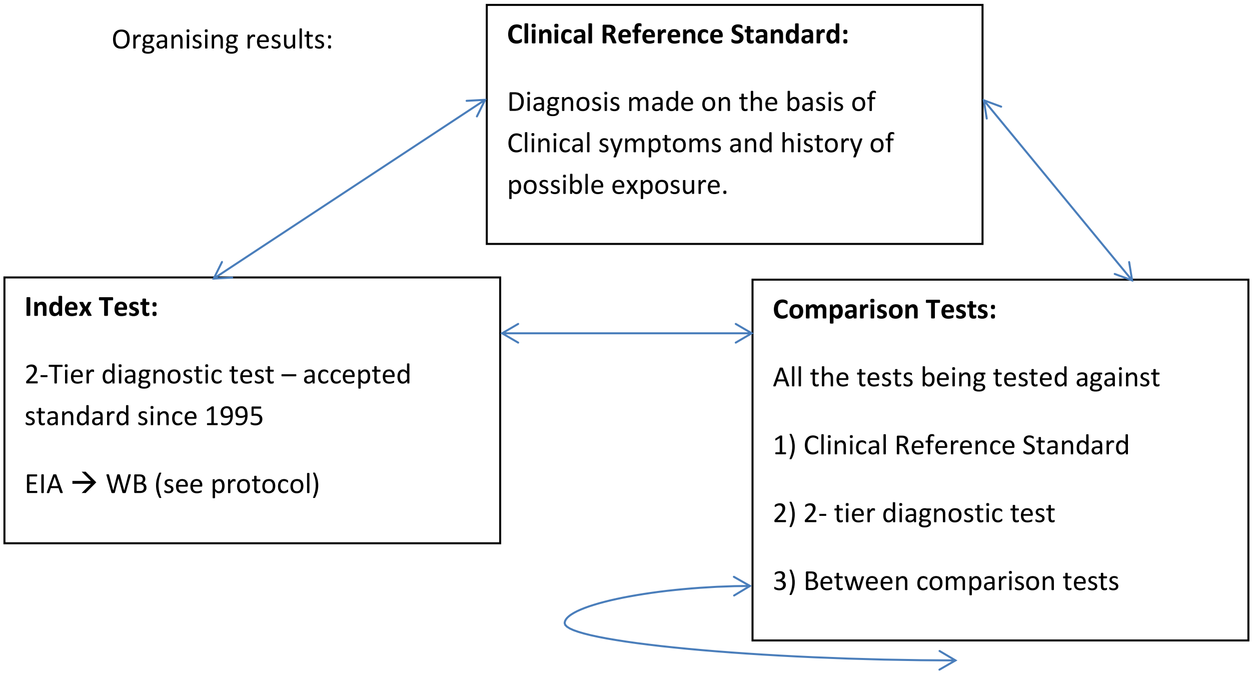

	D+ (CRS or Index)	D- (CRS or Index)
T+ (Index or comp test)	a	b
T- (Index or comp test)	c	d

Sn= a/a+c proportion with disease that test positive

Sp = d/b+d proportion without the disease that test negative

PPV= a/a+b (dependant on the sample) proportion of people with a positive result that actually have the disease. (post-test probability of having a disease.)

NPV= d/(c+d) Proportion with a negative result that do not have the disease. (Post-test probability of having the disease given a negative test =1-NPV)

The predictive values (PPV and NPV) are dependent on the prevalence in the population and are not directly transferable.

Data:

Sensitivity: Proportion of people with Lyme disease that test positive (index or comparison test) OR proportion of people that with Lyme index test positive that also test positive with the comparison test.

Specificity: proportion of people without Lyme disease that test negative (index or comparison test) OR proportion of people with a Lyme index test negative that also test negative with a comparison test.
