# Supplementary material for: The Accuracy of Diagnostic Tests for Lyme Disease in Humans, A Systematic Review and Meta-Analysis of North American Research
Source: PLoS One. 2016 Dec 21;11(12):e0168613. doi: 10.1371/journal.pone.0168613 (PMC5176185; doi:10.1371/journal.pone.0168613)
Supplement: S2 Text — (PDF) [file pone.0168613.s003.pdf]

S2: 48 Studies Included in the SR and a List of licensed tests for Lyme disease as of May 2015

48 Studies Included in the SR

1. Avery RA, Frank G, Eppes SC. Diagnostic utility of Borrelia burgdorferi cerebrospinal fluid polymerase chain reaction in children with Lyme meningitis. *The Pediatric infectious disease journal* 2005; 24: 705-708; doi:00006454-200508000-00010 [pii].

2. Bacon RM*, et al*. Serodiagnosis of Lyme disease by kinetic enzyme-linked immunosorbent assay using recombinant VlsE1 or peptide antigens of Borrelia burgdorferi compared with 2-tiered testing using whole-cell lysates. *The Journal of infectious diseases* 2003; 187: 1187-1199; doi:JID20849 [pii].

3. Branda JA*, et al*. 2-tiered antibody testing for early and late Lyme disease using only an immunoglobulin G blot with the addition of a VlsE band as the second-tier test. *Clinical infectious diseases : an official publication of the Infectious Diseases Society of America* 2010; 50: 20-26; doi:10.1086/648674 [doi].

4. Branda JA*, et al*. Two-tiered antibody testing for Lyme disease with use of 2 enzyme immunoassays, a whole-cell sonicate enzyme immunoassay followed by a VlsE C6 peptide enzyme immunoassay. *Clinical infectious diseases : an official publication of the Infectious Diseases Society of America* 2011; 53: 541-547; doi:10.1093/cid/cir464 [doi].

5. Brissette CA*, et al*. The borrelial fibronectin-binding protein RevA is an early antigen of human Lyme disease. *Clinical and vaccine immunology : CVI* 2010; 17: 274-280; doi:10.1128/CVI.00437-09 [doi].

6. Brunner M, Sigal LH. Use of serum immune complexes in a new test that accurately confirms early Lyme disease and active infection with Borrelia burgdorferi. *Journal of clinical microbiology* 2001; 39: 3213-3221.

7. Brunner M*, et al*. Immunoglobulin M capture assay for serologic confirmation of early Lyme disease: analysis of immune complexes with biotinylated Borrelia burgdorferi sonicate enhanced with flagellin peptide epitope. *Journal of clinical microbiology* 1998; 36: 1074-1080.

8. Burbelo PD*, et al*. Rapid, simple, quantitative, and highly sensitive antibody detection for lyme disease. *Clinical and vaccine immunology : CVI* 2010; 17: 904-909; doi:10.1128/CVI.00476-09 [doi].

9. Callister SM*, et al*. Ability of the borreliacidal antibody test to confirm lyme disease in clinical practice. *Clinical and diagnostic laboratory immunology* 2002; 9: 908-912.

10. Callister SM*, et al*. Sensitivity and specificity of the borreliacidal-antibody test during early Lyme disease: a "gold standard"? *Clinical and diagnostic laboratory immunology* 1996; 3: 399-402.

11. Embers ME*, et al*. Borrelia burgdorferi spirochetes that harbor only a portion of the lp28-1 plasmid elicit antibody responses detectable with the C6 test for Lyme disease. *Clinical and vaccine immunology : CVI* 2007; 14: 90-93; doi:CVI.00266-06 [pii].

12. Eshoo MW, *et al*. Direct molecular detection and genotyping of Borrelia burgdorferi from whole blood of patients with early Lyme disease. *PloS one* 2012; 7: e36825; doi:10.1371/journal.pone.0036825 [doi].

13. Fawcett PT, *et al*. Comparison of immunodot and western blot assays for diagnosing Lyme borreliosis. *Clinical and diagnostic laboratory immunology* 1998; 5: 503-506.

14. Gomes-Solecki MJ, *et al*. Recombinant chimeric Borrelia proteins for diagnosis of Lyme disease. *Journal of clinical microbiology* 2000; 38: 2530-2535.

15. Gomes-Solecki MJ, *et al*. Epitope length, genospecies dependency, and serum panel effect in the IR6 enzyme-linked immunosorbent assay for detection of antibodies to Borrelia burgdorferi. *Clinical and vaccine immunology : CVI* 2007; 14: 875-879; doi:CVI.00122-07 [pii].

16. Gomes-Solecki MJ, *et al*. A first-tier rapid assay for the serodiagnosis of Borrelia burgdorferi infection. *Archives of Internal Medicine* 2001; 161: 2015-2020; doi:ioi00721 [pii].

17. Gomes-Solecki MJ, *et al*. Recombinant assay for serodiagnosis of Lyme disease regardless of OspA vaccination status. *Journal of clinical microbiology* 2002; 40: 193-197.

18. Hilton E, Devoti J, Sood S. Recommendation to include OspA and OspB in the new immunoblotting criteria for serodiagnosis of Lyme disease. *Journal of clinical microbiology* 1996; 34: 1353-1354.

19. Jobe DA, *et al*. Significantly improved accuracy of diagnosis of early Lyme disease by peptide enzyme-linked immunosorbent assay based on the borreliacidal antibody epitope of Borrelia burgdorferi OspC. *Clinical and vaccine immunology : CVI* 2008; 15: 981-985; doi:10.1128/CVI.00079-08 [doi].

20. Johnson BJ, *et al*. Serodiagnosis of Lyme disease: accuracy of a two-step approach using a flagella-based ELISA and immunoblotting. *The Journal of infectious diseases* 1996; 174: 346-353.

21. Ledue TB, Collins MF, Craig WY. New laboratory guidelines for serologic diagnosis of Lyme disease: evaluation of the two-test protocol. *Journal of clinical microbiology* 1996; 34: 2343-2350.

22. Ledue TB, *et al*. Evaluation of the recombinant VlsE-based liaison chemiluminescence immunoassay for detection of Borrelia burgdorferi and diagnosis of Lyme disease. *Clinical and vaccine immunology : CVI* 2008; 15: 1796-1804; doi:10.1128/CVI.00195-08 [doi].

23. Liang FT, *et al*. Sensitive and specific serodiagnosis of Lyme disease by enzyme-linked immunosorbent assay with a peptide based on an immunodominant conserved region of Borrelia burgdorferi vlsE. *Journal of clinical microbiology* 1999; 37: 3990-3996.

24. Liveris D, *et al*. Improving the yield of blood cultures from patients with early Lyme disease. *Journal of clinical microbiology* 2011; 49: 2166-2168; doi:10.1128/JCM.00350-11 [doi].

25. Liveris D, *et al*. Comparison of five diagnostic modalities for direct detection of Borrelia burgdorferi in patients with early Lyme disease. *Diagnostic microbiology and infectious disease* 2012; 73: 243-245; doi:10.1016/j.diagmicrobio.2012.03.026 [doi].

26. Liveris D*, et al*. Quantitative detection of Borrelia burgdorferi in 2-millimeter skin samples of erythema migrans lesions: correlation of results with clinical and laboratory findings. *Journal of clinical microbiology* 2002; 40: 1249-1253.

27. Magnarelli LA*, et al*. Use of recombinant antigens of Borrelia burgdorferi in serologic tests for diagnosis of lyme borreliosis. *Journal of clinical microbiology* 1996; 34: 237-240.

28. Marques AR*, et al*. Detection of immune complexes is not independent of detection of antibodies in Lyme disease patients and does not confirm active infection with Borrelia burgdorferi. *Clinical and diagnostic laboratory immunology* 2005; 12: 1036-1040; doi:12/9/1036 [pii].

29. Mogilyansky E*, et al*. Comparison of Western immunoblotting and the C6 Lyme antibody test for laboratory detection of Lyme disease. *Clinical and diagnostic laboratory immunology* 2004; 11: 924-929; doi:10.1128/CDLI.11.5.924-929.2004 [doi].

30. Nocton JJ*, et al*. Detection of Borrelia burgdorferi DNA by polymerase chain reaction in cerebrospinal fluid in Lyme neuroborreliosis. *The Journal of infectious diseases* 1996; 174: 623-627.

31. Nowakowski J*, et al*. Blood cultures for patients with extracutaneous manifestations of Lyme disease in the United States. *Clinical infectious diseases : an official publication of the Infectious Diseases Society of America* 2009; 49: 1733-1735; doi:10.1086/648076 [doi].

32. Pavia CS*, et al*. An indirect hemagglutination antibody test to detect antibodies to Borrelia burgdorferi in patients with Lyme disease. *Journal of microbiological methods* 2000; 40: 163-173; doi:S0167-7012(00)00119-6 [pii].

33. Phillips SE*, et al*. A proposal for the reliable culture of Borrelia burgdorferi from patients with chronic Lyme disease, even from those previously aggressively treated. *Infection* 1998; 26: 364-367.

34. Porwancher RB*, et al*. Multiplex immunoassay for Lyme disease using VlsE1-IgG and pepC10-IgM antibodies: improving test performance through bioinformatics. *Clinical and vaccine immunology : CVI* 2011; 18: 851-859; doi:10.1128/CVI.00409-10 [doi].

35. Qiu B*, et al*. Selection of continuous epitope sequences and their incorporation into poly(ethylene glycol)-peptide conjugates for use in serodiagnostic immunoassays: application to Lyme disease. *Biopolymers* 2000; 55: 319-333; doi:10.1002/1097-0282(2000)55:4<319::AID-BIP1005>3.0.CO;2-W [pii].

36. Sapi E*, et al*. Improved culture conditions for the growth and detection of Borrelia from human serum. *International journal of medical sciences* 2013; 10: 362-376; doi:10.7150/ijms.5698 [doi].

37. Sikand VK, Rothel JS, Martin RM. Diagnosis of Lyme borreliosis by a whole-blood gamma interferon assay for cell-mediated immune responses. *Clinical and diagnostic laboratory immunology* 1999; 6: 445.

38. Sivak SL*, et al*. Accuracy of IgM immunoblotting to confirm the clinical diagnosis of early Lyme disease. *Archives of Internal Medicine* 1996; 156: 2105-2109.

39. Steere AC, *et al*. Prospective study of serologic tests for lyme disease. *Clinical infectious diseases : an official publication of the Infectious Diseases Society of America* 2008; 47: 188-195; doi:10.1086/589242 [doi].

40. Tilton RC, Sand MN, Manak M. The western immunoblot for Lyme disease: determination of sensitivity, specificity, and interpretive criteria with use of commercially available performance panels. *Clinical infectious diseases : an official publication of the Infectious Diseases Society of America* 1997; 25 Suppl 1: S31-4.

41. Trevejo RT, *et al*. Evaluation of two-test serodiagnostic method for early Lyme disease in clinical practice. *The Journal of infectious diseases* 1999; 179: 931-938; doi:JID980677 [pii].

42. Wieneke CA, *et al*. Evaluation of whole-cell and OspC enzyme-linked immunosorbent assays for discrimination of early lyme borreliosis from OspA vaccination. *Journal of clinical microbiology* 2000; 38: 313-317.

43. Wormser GP, *et al*. Comparison of the yields of blood cultures using serum or plasma from patients with early Lyme disease. *Journal of clinical microbiology* 2000; 38: 1648-1650.

44. Wormser GP, *et al*. Effect of Borrelia burgdorferi genotype on the sensitivity of C6 and 2-tier testing in North American patients with culture-confirmed Lyme disease. *Clinical infectious diseases : an official publication of the Infectious Diseases Society of America* 2008; 47: 910-914; doi:10.1086/591529 [doi].

45. Wormser GP, *et al*. Improving the yield of blood cultures for patients with early Lyme disease. *Journal of clinical microbiology* 1998; 36: 296-298.

46. Wormser GP, *et al*. Single-tier testing with the C6 peptide ELISA kit compared with two-tier testing for Lyme disease. *Diagnostic microbiology and infectious disease* 2013; 75: 9-15; doi:10.1016/j.diagmicrobio.2012.09.003 [doi].

47. Yu Z, *et al*. Presentation of peptide antigens as albumin conjugates for use in detection of serum antibodies by enzyme-linked immunosorbent assay. *Bioconjugate chemistry* 1996; 7: 338-342; doi:10.1021/bc960018s [doi].

48. Yu Z, *et al*. Multi-well ELISA based on independent peptide antigens for antibody capture. Application to Lyme disease serodiagnosis. *Journal of immunological methods* 1996; 198: 25-33; doi:0022175996001408 [pii].

List of licensed tests for Lyme disease as of May 2015

Name in Tables	Test Name	Company	Description/ Target	SR Table	FDA approved	HCMDA approved
EIA						
	3M IGG Fastlyme(TM) Test	3M Company			yes	
	Bartels lyme disease IgM EIA	Baxter Diagnostics Inc.			yes	
Vidas	Vidas Lyme Screen II (IgM and IgG)	Biomerieux Vitek Inc.	Whole cell sonicate, objective reading.	T2, T3, T5	yes	yes
BION Borrelia Burgdorferi Antigen Substrate Slide	BION Borrelia Burgdorferi Antigen Substrate Slide	MBL international	Whole-cell sonicated B. burgdorferi B31. Semi-quantitative.	T6	yes	
	Platelia® Lyme IgM /IgG	Bio-Rad Laboratories	Whole-cell sonicated B. burgdorferi B31 IgG and IgM. Objective reading.		yes	
	Boston Biomedica Inc. Borrelia burgdorferi IgM/ IgG	Boston Biomedica Inc.			yes	
Cambridge	Human Lyme EIA for the detect of antibodies	Cambridge Bioscience Corp.	Whole-cell sonicated B. burgdorferi B31 IgG and IgM. Objective reading.	T2, T6	removed	
	Prevue™ *Borrelia* burgdorferi antibody detection assay	Chembio Diagnostic Systems Inc.	Prevue™ *Borrelia* burgdorferi antibody detection assay uses recombinant antigens: OspA, OspB, OspC (3 types), p 93 and flagellin. Objective reading.		yes	
	IS *Borrelia burgdorferi* IgG/ IgM ELISA test system	Columbia Bioscience Inc.			yes	
	Lyme IgG/IgM ELISA	Diagnostic Automation	Whole-cell sonicated B. burgdorferi B31 IgG and IgM. Objective reading.			yes
	Lyme-check ™ test kit	Diagnostic Technology Inc.			yes	
	Lyme Disease microassay (IgM/IgG)	Diamedix corp.			yes	
Liason	Liason Borrelia IgG /IgM assay model 310870 (CLIA)	Diasorin	chemiluminescence assay, quantitative determination IgG=VlsE target, IgM= two recombinant antigens OspC and	T2, T3, T6		yes

			VlsE. Objective reading		
	Anti-B. burgdorferi VlsE IgG	Euroimmun	Antigens: extract of Borrelia burgdorferi sensu stricto, Borrelia garinii and Borrelia afzelii (whole antigen) / recombinant VlsE from Borrelia burgdorferi sensu stricto (IgG). Objective reading		yes
	Anti-Borrelia VlsE IgG	Euroimmun	Antigens: extract of Borrelia burgdorferi sensu stricto, Borrelia garinii and Borrelia afzelii (whole antigen) / recombinant VlsE from Borrelia burgdorferi sensu stricto (IgG). Objective reading		yes
	Lyme Screen IgG/IgM	Euroimmun	Recombinant VlsE from Borrelia burgdorferi sensu stricto and Borrelia OspC. Semiquantitative. Objective reading		yes
	CSF - Anti-Borrelia VlsE IgM/ IgG	Euroimmun	Whole antigen, SDS extract of Borrelia burgdorgeri sensu stricto, B. garingii and B. afzelii plus recombinant VlsE of B. burgdorferi sensu stricto.		yes
	Anti-Borrelia Select IgM/ IgG	Euroimmun	Highly specific recombinant antigens from different human pathogenic Borrelia strains are used as the antigen. The test includes VlsE. Objective Reading.		yes
	Lyme IgM ELISA test	Gull laboratories Inc.	Whole cell sonicate B. burgdorferi (JD1 strain)	yes	
	Lyme Disease ELISA (IgG)	Hillcrest Biologicals		yes	
	Lyme disease IFA (IgG/ IgM)	Hillcrest Biologicals		yes	
	Borrelia + VlsE IgG	IBL International	Sandwich ELISA IgG= mixed antigens for B. burgdorferi, B. garinii and B. afzelii combined		yes

			with a recombinant VlsE antigen.			
	Borrelia 14kDa & OspC IgM	IBL International	Sandwich ELISA IgM= recombinant 14kDa and native OspC (from B. afzelii).			Yes
Immunetics C6	Immunetics® C6 *B. burgdorferi* ELISA™ kit	Immunetics®	Recombinant protein. Objective reading.	T2, T3, T6	yes	yes
	Quanta Lite® Lyme *B. burgdorferi* IgG/IgM ELISA kit	Inova diagnostics Inc.			yes	
MarDx Lyme disease EIA (IgM/IgG) test system	MARDX Lyme disease EIA (IgG & IgM) test system	MARDX diagnostics Inc.	Whole cell sonicate B. burgdorferi (Strain B 31) ,objective reading	T3, T6	yes	yes
	MARDX Lyme disease IgG/IgM IFA test system	MARDX diagnostics Inc.	Whole cell sonicate B. burgdorferi (strain B31). objective reading		yes	
	Borrelia burgdorferi IgM antibody test system	Medical Diagnostic Technologies Inc.			yes	
	Borrelia burgdorferi polyval IgG/IgM antibody test	Medical diagnostic technologies Inc.			yes	
	RecomWell Borrelia IgM/ IgG	Mikrogen	Immunodominant antigens of B. burgdorferi sensu stricto, B. garinii and B. afzelii. IgM: OspC, p41/internal, VlsE and IgG: p100, OspC, VlsE, p18			yes
	Classic B. burgdorferi IgG/ IgM	Serion	Qualitative and quantitative immunoassays. Mixture of native antigens from B. afzelii and B. garinii and recombinant VlsE.			yes
	Borrelia burgdorferi IgG/ IgM ELISA test system	Trinity Biotech USA	Whole cell sonicate of B. burgdorferi (strain B31)		yes	
	Lyme-Spot IF kit	Vitek systems Inc.			yes	
Wampole	*Borrelia burgdorferi* IgG/IgM ELISA test system	Wampole laboratories	Whole cell sonicate. Objective reading.	T2, T3, T5	yes	
	FIAX test kit for Lyme Disease antibodies (IgG/IgM)	Whittaker Bioproducts Inc.			yes	
Lyme STAT test	Lyme STAT M or G test kit (IgG/IgM)	Whittaker Bioproducts Inc.	Whole cell sonicate. Subjective	T3, T6	yes	

kit			reading.			
Zeus AtheNA	Athena multi-lyte borrelia VlsE-1/PEPC10 plus test system	Zeus Scientific Inc.	VlsE-1/ PEPC10- recombinant multiplex IgG. Objective reading	T2	yes	
	IFA test for antibodies to *Borrelia burgdorferi*	Zeus Scientific Inc.	Semi-quantitative. Whole cell sonicate, B. burgdorferi (B31 Strain).		yes	Yes
Zeus ELISA	Lyme ELISA test system (IgG/IgM)	Zeus Scientific Inc.	Whole cell sonicate, B. burgdorferi (B31 Strain). Objective Reading.	T2	yes	yes
	Zeus ELISA Borrelia VlsE-1/PEPC10 IgG/IgM test system	Zeus Scientific Inc.	Inactivated VlsE-1 and pepC10 antigens. Objective reading		yes	yes
**Westernblot**						
BBI Bb IgM or IgG Western Blot Test Kit	BBI-Biotech research laboratories *B. burgdorferi* IgM Western blot kit	Boston Biomedica Inc.	Purified antigen. Subjective reading	T4, T6	yes	
Cambridge Biotech Human Lyme IgG /IgM Western Blot	Cambridge Biotech Human Lyme IgG / IgM western blot (90111/ 90112)	Cambridge Biotech Corp. / Calypte Biomedical Corp.		T6	yes	
	Human Lyme IgM/IgG western blot kit	Cambridge Diagnostics Ireland Ltd.			yes	
	Anti-B. burgdorferi US EuroLine IgG /IgM	Euroimmun	Whole antigen, SDS extract of B. burgdorferi sensu stricto			Yes
	Anti-B. afzelii IgG	Euroimmun	Whole antigen, SDS extract of B. afzelii			Yes
	Anti-B. garinii IgG	Euroimmun	Whole antigen, SDS extract of or *B. garinii*			yes
ImmunoDOT Borrelia Dot Blot IgG/IgM Test (GenBio)	ImmunoDOT Borrelia dot blot G or M test	GenBio	whole B. burgdorferi antigens and four specific proteins: a high molecular weight protein (HMW), flagellin, a 39 kD protein (p39/ BmpA), and outer surface protein C (OspC). Subjective reading.	T4, T6	yes	
Immunowell Borrelia (Lyme) test	Immunowell *Borrelia* (Lyme) test	GenBio	Whole cell B. burgdorferi	T6	yes	

	Borrelia burgdorferi B31 IgM/IgG line blot test	Gold Standard diagnostics	Target proteins IgG=p83/93/100 (Late LD) Oms66 (p66), OppA-2 (p58), Heat Shock (p45), Flagellin (p41), BmpA (p39). OspA (p31), (p30, p28, p18), OspC (p23). IgM= flagellin (p41), and OspC (p23). Objective reading.		yes	
	Qualicode *B. burgdorferi* gG/IgM western blot kit models DK-C052-024/DK-C062-024	Immunetics ®			yes	
Marblot	Mardx Lyme Disease (IgM/IgG) Marblot strip test system	Mardx Diagnostics Inc.	Purified antigens/ Subjective reading.	T2, T4, T5, T6	yes	yes
	RecomLine Borrelia IgM/ IgG	Mikrogen	Detects antibodies to B. burgdorferi, B. garinii, B. afzelii, B. spielmanii and B. bavariensis targeting VlsE, OspC and p18 (DbpA), p58 (B. garinii), p39 (B. afzelii), OspA (B. afzelii), p100 (B. afzelii), p41 (B. burgdorferi ss)			yes
MRL diagnostics: Lyme Disease Bb genogroup 1 WB IgG or IgM	Lyme disease *B. burgdorferi* genotype 1 western blot IgG/IgM (WB0400G/ WB0400M)	MRL Diagnostics		T5	yes	
Virablot	Viramed biotech *Borrelia* B31 IgM/IgG Virablot	Viramed Biotech Ag	Purified antigen/ Subjective reading.	T2, T4	yes	
	Viramed Biotech AG *Borrelia* B31 IgG /IgM Virastripe® Model V-BBSGUS	Viramed Biotech Ag	Native, purified antigens B. burgdorferi (strain B31), Subjective reading.		yes	
	Borrelia burgdorferi IgG or IgM western blot test system	Zeus Scientific Inc.			yes	
**PCR**						
	Borrelia Burgdorferi Primer Pair	Focus Diagnostics				
